# Aggregation‐Induced Resonance Energy Transfer in Polymer Dots to Boost Electrochemiluminescence Performance for Bioimaging of Glycan on Cells

**DOI:** 10.1002/advs.202524265

**Published:** 2026-01-22

**Authors:** Chao Wang, Mengjiao Li, Yiran Li, Xiangfu Hu, Chunlan Li, Ningning Wang, Huangxian Ju

**Affiliations:** ^1^ State Key Laboratory of Analytical Chemistry For Life Science School of Chemistry and Chemical Engineering Nanjing University Nanjing China; ^2^ School of Pharmacy Binzhou Medical University Yantai China; ^3^ Henan Key Laboratory of Biomolecular Recognition and Sensing Henan Joint International Research Laboratory of Chemo/Biosensing and Early Diagnosis of Major Diseases College of Chemistry and Chemical Engineering Shangqiu Normal University Shangqiu China

**Keywords:** aggregation, cell imaging, electrochemiluminescence, resonance energy transfer, thermally activated delayed fluorescence

## Abstract

To break through the bottleneck of electrochemiluminescence (ECL) efficiency (*Φ_ECL_*), this study pioneers an aggregation‐induced resonance energy transfer (ARET) mechanism for simultaneously boosting the exciton utilization efficiency and photoluminescence quantum yield of ECL emitters. This mechanism is achieved by synergistically integrating aggregation‐induced emission (AIE), resonance energy transfer (RET), and thermally activated delayed fluorescence (TADF) within triethylamine‐conjugated polymers featuring an AIE‐active fluorene derivative as energy donor and a typical TADF molecule (DMAC‐TRZ) as acceptor. Spectroscopic and theoretical analyses confirm that the polymers exhibit efficient RET, unique ARET behavior, and intrinsic TADF property with an ultralow singlet‐triplet energy gap. The optimal coreactant‐containing polymer dots yield self‐enhanced ECL with an *Φ_ECL_* of 92.6%, significantly outperforming those of the equimolar [Ru(bpy)_3_]^2^^+^ and state‐of‐the‐art organic nanomaterials. Leveraging the small size, low ECL potential of +0.96 V, high *Φ_ECL_*, and minimal cytotoxicity of the polymer dots, a hydrazide‐functionalized ECL probe is developed for sensitive ECL imaging of cell surface glycans, offering improved signal‐to‐background ratio over fluorescence‐based methods. The proposed ARET mechanism provides a transformative paradigm for designing efficient ECL nanoemitters and bioimaging protocols.

## Graphical abstract

This study pioneers an aggregation‐induced resonance energy transfer mechanism by synergistically integrating aggregation‐induced emission (AIE), resonance energy transfer (RET), and thermally activated delayed fluorescence (TADF) within co‐reactant conjugated polymers to develop self‐enhanced ECL emitters, which achieves an ECL efficiency up to 92.6% for sensitive ECL bioimaging.

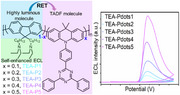

## 1 Introduction

Electrochemiluminescence (ECL) stands out as a powerful analytical technique with high sensitivity and minimal background interference [1], facilitating groundbreaking applications in single‐molecule detection [2, 3, 4], single‐cell analysis [5, 6], nanomaterial‐based electrocatalysis study [7, 8], and multilevel information encryption [9, 10]. The performance of ECL analysis relies heavily on the emission efficiency of ECL emitter (*Φ_ECL_*) [11, 12], which is critically influenced by the exciton utilization efficiency and photoluminescence quantum yield [13, 14]. The phosphorescent [Ru(bpy)_3_]^2^^+^ remains the benchmark ECL emitter due to its near‐unity harvesting of both electrogenerated singlet and triplet excitons for ECL emission [15]. However, [Ru(bpy)_3_]^2^^+^−based nanoemitters face significant challenges in positive cellular ECL imaging, including leakage‐induced background, exogenous coreactant‐induced cytotoxicity, and steric‐hindrance‐impaired targeting [3, 4, 5]. These challenges drive the need to develop compact and biocompatible self‐enhanced ECL nanoemitters with an *Φ_ECL_* comparable to [Ru(bpy)_3_]^2^^+^ for bioimaging and bioanalysis applications.

Polymer dots (Pdots), as sub‐30 nm nanostructures derived from conjugated fluorescent polymers, possess exceptional photophysical characteristics and robust stability [16, 17], and have been known as compelling ECL emitters for bioanalysis. Therefore, various strategies have been designed to boost the *Φ_ECL_* of Pdots [18, 19, 20]. For example, aggregation‐induced emission (AIE)‐active Pdots were synthesized to improve the quantum yield of Pdots with tetraphenylethene‐based monomer [18]. Our previous work developed three‐component Pdots to improve the ECL performance via dual intramolecular resonance energy transfer (RET), but both complex synthesis and difficulty in precisely controlling the component ratios hinder their further development [19]. We then designed coreactant‐embedded Pdots to greatly boost the *Φ_ECL_* via dual intramolecular electron transfer [20]. Critically, these fluorescent Pdots are fundamentally limited by inefficient exciton harvesting due to spin‐forbidden transitions [21], which impedes further enhancement of *Φ_ECL_*. To transcend the spin limitations, some emitters, such as thermally activated delayed fluorescence (TADF) emitters [22, 23], have been designed to achieve near‐unity exciton utilization by efficient reverse intersystem crossing (RISC) [24]. Herein, we proposed an aggregation‐induced RET (ARET) mechanism to boost the *Φ_ECL_* of Pdots by synergistically integrating AIE, RET, and TADF within triethylamine coreactant‐conjugated polymers (TEA‐Ps), which led to an *Φ_ECL_* of 92.6% at a relatively low anodic potential.

**FIGURE 1:**
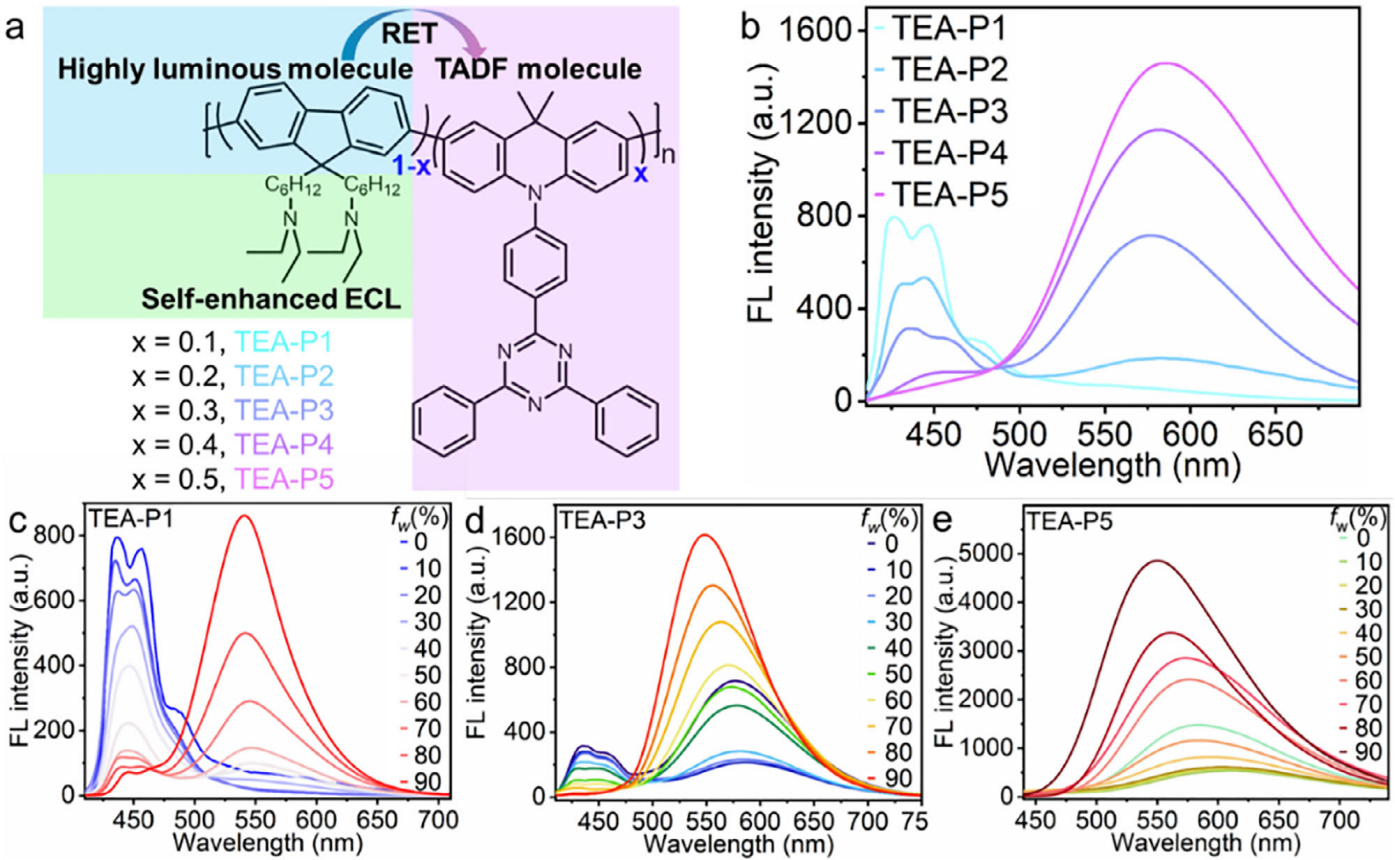
(a) Schematic illustration of TEA‐Ps. (b) FL spectra of TEA‐P1∼TEA‐P5 in THF. (c–e) FL spectra of TEA‐P1, TEA‐P3, TEA‐P5 in THF/water mixture with different water fractions (*f_w_*). *λ*_ex_ = 300 nm.

The ARET mechanism was achieved by using AIE‐active 6,6’‐(9*H*‐fluorene‐9,9‐diyl)bis(*N*,*N*‐diethylhexan‐1‐amine) as energy donor for improving the quantum yield and self‐enhanced ECL, and a typical TADF molecule, DMAC‐TRZ, as energy acceptor for efficient exciton harvesting (Figure 1a) [25]. By systematically controlling the molar fraction of energy acceptor (x, where 1‐x is the donor fraction) within the range of 0.1 to 0.5, we aimed to achieve the most efficient RET process, thereby maximizing the overall ECL performance. The RET feasibility, ARET behavior, and TADF property of TEA‐Ps were well demonstrated by both experimental results and density functional theory calculations. The corresponding Pdots (TEA‐Pdots), functioned as self‐enhanced ECL emitters, showed dramatically enhanced ECL intensity. Leveraging the small size, low anodic ECL peak potential, high *Φ_ECL_*, and minimal cytotoxicity, a compact ECL probe was prepared by coupling the TEA‐Pdots with adipic dihydrazide (ADH) for sensitive ECL imaging of glycans on live cells. This study pioneers an ARET enhancement strategy with a RET system comprising a highly‐luminous AIE donor and an efficient exciton‐utilizing TADF acceptor, advancing the developments and bioanalysis applications of organic ECL emitters.

## 2 Results and Discussion

### 2.1 Aggregation‐Induced Resonance Energy Transfer of TEA‐Ps

The triethylamine‐conjugated polymers, TEA‐P1∼TEA‐P5 with progressively increasing DMAC‐TRZ ratio, were synthesized via Suzuki coupling reaction (Scheme S1) and verified by ^1^H nuclear magnetic resonance spectroscopy (Appendixes S1–S14). The center‐to‐center distance between the energy donor and acceptor was measured to be 10.82 Å (Figure S1a), which was within the effective distance range for RET [26]. The fluorescence (FL) emission of fluorene peaking at 402 nm showed significant overlap with the absorption of DMAC‐TRZ peaking at 383 nm (Figure S1b), while their FL excitation wavelengths (Figure S1c) collectively satisfied the criteria for RET [27]. Upon excitation of fluorene at 300 nm, the FL spectra of TEA‐P1∼TEA‐P5 (Figure 1b) exhibited gradually decreased emission of the donor at the range of 410–490 nm, accompanied by an increasing emission of prominent acceptor at 580 nm, confirming efficient RET from fluorene derivative to DMAC‐TRZ. Notably, compared to the emission peak of DMAC‐TRZ at 571 nm (Figure S1b), the corresponding emission peaks of TEA‐Ps red‐shifted slightly (Figure 1b), likely due to the enhanced conjugation within the polymer backbone. Intriguingly, the FL spectra of TEA‐Ps in tetrahydrofuran (THF)/water mixtures with increasing water fractions (*f_w_*), as exemplified by TEA‐P1, TEA‐P3, and TEA‐P5 (Figure 1c–e), exhibited a progressively decreased emission of the donor, concomitant with a marked emission enhancement of the acceptor. Although fluorene derivatives exhibited AIE characteristics [28], the FL emission of donor decreased with increasing *f_w_*, while the FL emission of acceptor (related to DMAC‐TRZ) continuously intensified (Figure S2a,c,e), suggesting that the emission enhancement of the acceptor arose from the synergistic effects of AIE and RET. Moreover, as *f_w_* increased, the FL intensity of isolated DMAC‐TRZ initially decreased and then increased when *f_w_* exceeded 50% (Figure S3a,b). We termed this interesting phenomenon as “aggregation‐induced resonance energy transfer (ARET)”. Additionally, the increase of *f_w_* also resulted in continuous hypsochromic shift of the acceptor‐related FL emission (Figures S2b,d,f, and S3c), attributed to the aggregation of TEA‐Ps into nanostructures, which suppressed non‐radiative decay by restricting intramolecular motions [29]. Meanwhile, more than ten‐fold enhancement in the quantum yield of TEA‐P5 was observed as *f_w_* rose from 0% to 90% (Table S1), demonstrating that ARET was an effective strategy to boost the quantum yield of ECL emitters.

### 2.2 TADF Property of TEA‐Ps

**FIGURE 2:**
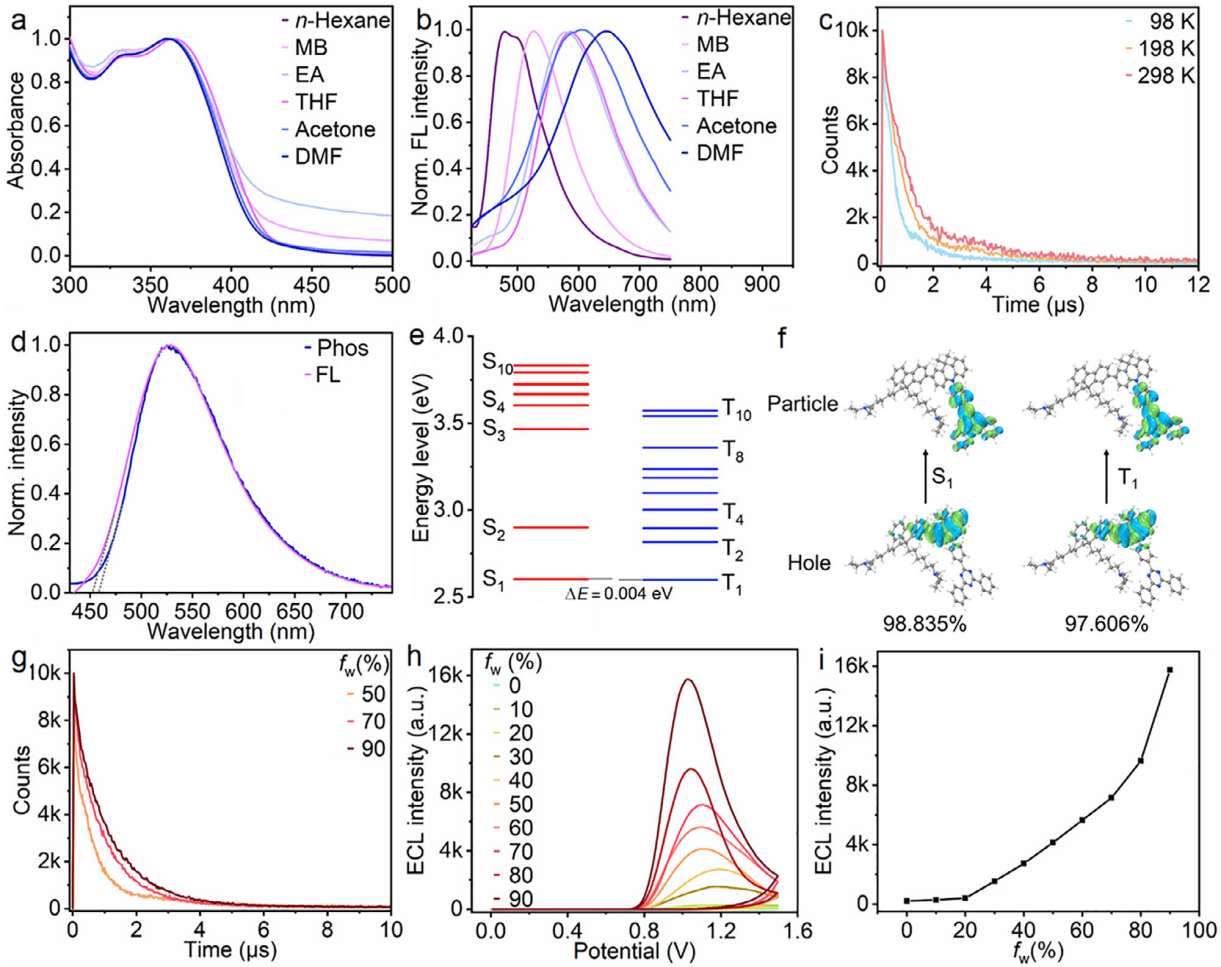
(a) UV–vis absorption and (b) normalized FL spectra of TEA‐P5 in different solvents. (c) Temperature‐dependent photoluminescence decay and (d) FL and phosphorescence (Phos, 77 K) spectra of TEA‐P5 in toluene. *λ*_ex_ = 300 nm. (e) Energy diagram of excited states of TEA‐P5, including the lowest ten singlet and triplet excited states at M06‐2x(D3)/def2‐TZVP level. (f) Natural transition orbitals of TEA‐P5 in S_1_ and T_1_ states (the percentages represent transition possibility, green and blue regions denote the positive and negative orbital phases, respectively). (g) Transient photoluminescence decay curves of TEA‐P5 in THF/water mixtures with different *f_w_*. (h) ECL‐potential curves of TEA‐P5 or its aggregates modified GCE in 0.1 m PBS (pH 7.4). Here TEA‐P5 and its aggregates were dispersed in THF/water mixture with different *f_w_* for electrode modification. (i) Plot of ECL intensity vs *f_w_*.

With the incorporation of DMAC‐TRZ, the TADF properties of TEA‐Ps were systematically investigated with TEA‐P5 serving as the representative due to its strongest FL emission. Its UV–vis absorption and FL spectra in different polar solvents (Figure 2a,b) revealed distinct solvatochromism, indicating a charge‐transfer (CT) excited state [30]. TEA‐P5 demonstrated a prompt FL lifetime of 25 ns and a delayed FL lifetime of 2.1 µs at 298 K, and the delayed FL lifetime shortened with the lowering temperature (Figure 2c), aligning with TADF behavior [31]. Based on the onsets of FL and phosphorescence spectra (Figure 2d), the first excited singlet state (S_1_) and triplet state (T_1_) energies of TEA‐P5 were estimated to be

2.731 and 2.713 eV respectively, corresponding to a remarkably small energy gap (*ΔE*_ST_) of 0.0179 eV [32]. This narrow gap could facilitate the transfer of T_1_ excitons to S_1_ via RISC, which was further corroborated by theoretical calculations predicting an even smaller *ΔE*_ST_ of 0.004 eV (Figure 2e; Table S2). Natural transition orbital simulations confirmed spatially separated hole‐particle distributions in both S_1_ and T_1_ states of TEA‐P5 monomer (Figure 2f), consistent with CT characteristics [30]. Additionally, the delayed FL lifetime of TEA‐P5 was prolonged as *f_w_* increased from 50% to 90% (Figure 2g), likely due to aggregation‐induced suppression of non‐radiative decay [33]. Interestingly, upon coating the aggregates of TEA‐P5 on the electrode, the ECL emission intensified as the *f_w_* increased from 0% to 90% (Figure 2h,i), implying that the aggregation of TEA‐P5 effectively amplified its ECL emission.

### 2.3 Characterization of TEA‐Pdots

**FIGURE 3:**
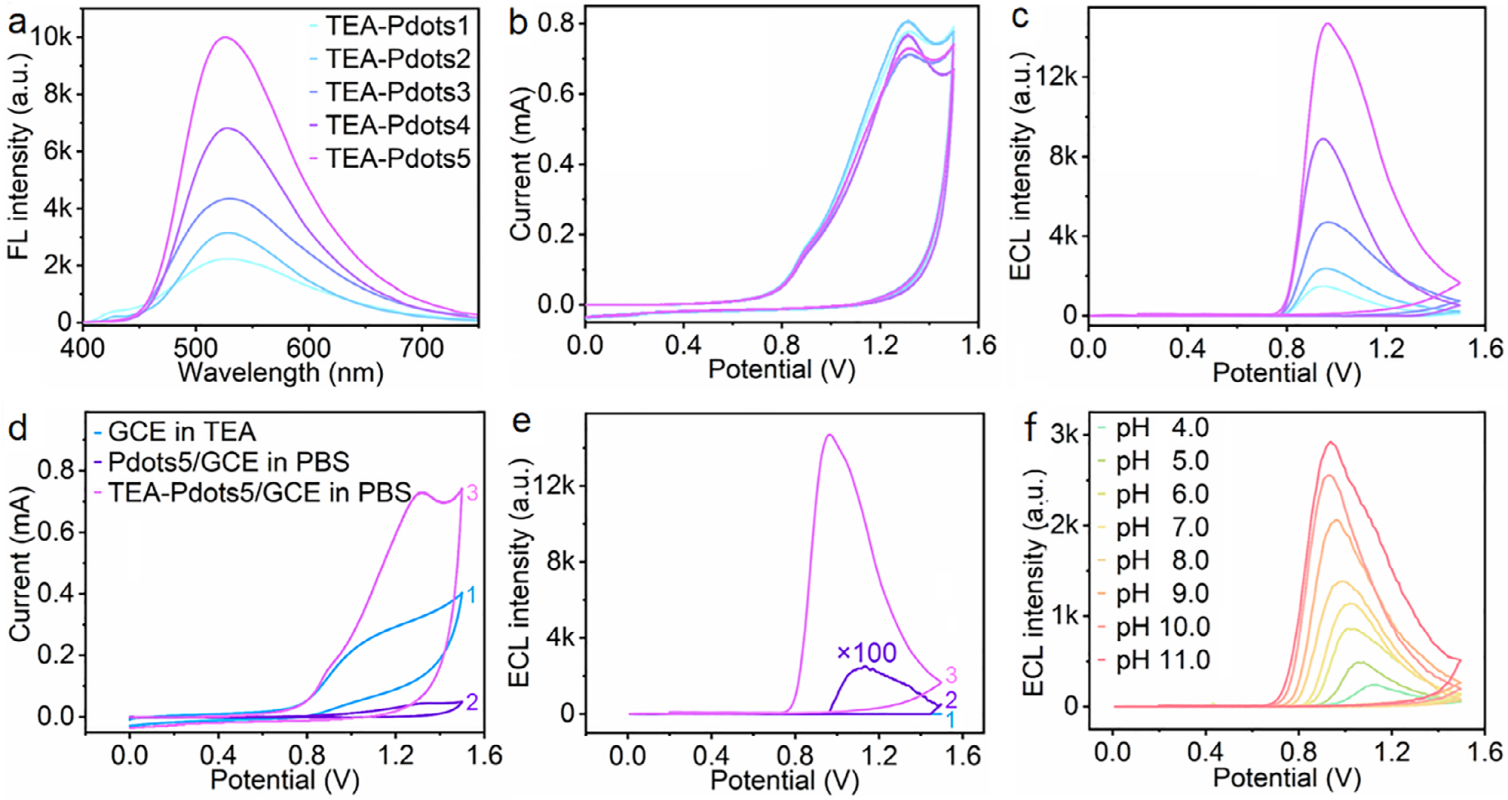
(a) FL spectra of TEA‐Pdots1∼TEA‐Pdots5. λex = 300 nm. (b) CV and (c) ECL curves of TEA‐Pdots1∼TEA‐Pdots5 modified GCE in 0.1 m PBS (pH 7.4). PMT = 400 V. (d) CV and (e) ECL curves of GCE (1), Pdots5/GCE (2), and TEA‐Pdots5/GCE (3) in 0.1 m PBS (pH 7.4) in presence (a) and absence (b, c) of 10 mm TEA. PMT = 400 V. (f) ECL curves of TEA‐Pdots5/GCE in 0.1 m PBS with different pHs. PMT = 300 V.

By reprecipitating TEA‐P1∼TEA‐P5 or the polymers without tertiary amine modification (P1∼P5) with amphiphilic poly(styrene‐ co‐maleic anhydride) (PSMA) [34], TEA‐Pdots1∼TEA‐Pdots5 and Pdots1∼Pdots5 were obtained, respectively. The TEA‐Pdots were predominantly spherical with a diameter of 7 nm, and no significant morphological change was observed upon increasing DMAC‐TRZ ratio (Figures S4 and S5). The Zeta potential analysis of TEA‐Pdots1∼TEA‐Pdots5 displayed a positive charge shift along with progressively declining surface charge as the content of donor decreased (Figure S6a), confirming the existence of tertiary amine groups in TEA‐Pdots. The X‐ray photoelectron spectrum of TEA‐Pdots5 showed a peak characteristic of protonated tertiary amine at 402.2 eV (Figure S6b), directly evidencing amine protonation. The FL emission peak of TEA‐Pdots was observed at 530 nm (Figure 3a), closing to the FL emission peak of TEA‐Ps at *f_w_* = 90% (Figure S2f). Notably, the FL emission of TEA‐Pdots intensified gradually with the acceptor fraction, peaking at TEA‐Pdots5 with a 4.5‑fold enhancement over TEA‐Pdots1. This enhancement arose not merely from the higher acceptor content, but from an optimal synergy in which efficient RET maximizes the high luminance of the AIE donor and the exciton‑harvesting ability of the TADF acceptor. Different from TEA‐Ps in THF (Figure 1b), only TEA‐Pdots1 and TEA‐Pdots2 showed fluorene‐associated FL emission peaks at 400–450 nm (Figure 3a), confirming the ARET effect of TEA‐Pdots.

### 2.4 Electrochemical and ECL Behaviors of TEA‐Pdots

The CV profiles of TEA‐Pdots1∼TEA‐Pdots5 all exhibited two oxidation peaks at +0.90 and +1.30 V, which led to ECL signals with onset at +0.75 and peak at +0.96 V (Figure 3b and 3c). Among the TEA‐Pdots, TEA‐Pdots5 exhibited the highest ECL emission, achieving a 10‐fold enhancement in ECL intensity compared to TEA‐Pdots1. While Pdots1∼Pdots5 exhibited exogenous TEA concentration‐dependent ECL enhancement (Figure S7a), supplemental TEA addition hardly affected the ECL responses of TEA‐Pdots1∼TEA‐Pdots5 (particularly TEA‐Pdots1 and 2) (Figure S7b), indicating self‐enhanced ECL generation and the successful integration of tertiary amine groups as built‐in coreactants within TEA‐Pdots.

The ECL properties of TEA‐Pdots were further elucidated with TEA‐Pdots5. Upon the anodic scan, the first oxidation peak occurred at +0.90 V with an onset potential of +0.70 V, aligned closely with that of TEA (Figure 3d, curve 1 and 3), indicating that the tertiary amine in TEA‐Pdots5 was first oxidized to generate TEA^•+^‐Pdots5. The second oxidation peak occurred at +1.30 V, close to the oxidation potential of Pdots5 (Figure 3d, curve 2 and 3). Notably, TEA‐Pdots5 started to generate ECL emission at +0.72 V and reached its maximum value at +0.96 V, while Pdots5 just began to oxidize at +0.96 V (Figure 3e, curve 2 and 3), verifying that the ECL emission originated from the oxidation of tertiary amine within TEA‐Pdots5 at a relatively low oxidation potential (LOP) [35]. Moreover, as the pH of PBS changed from 4 to 11, TEA‐Pdots5 exhibited a pH‐dependent ECL enhancement and peak potential shift (Figure 3f; Figure S8a), resulting from the facilitated deprotonation of TEA^•+^‐Pdots5. Whereas, the ECL intensity of Pdots5 decreased as the pH increased from 7 to 11 (Figure S8b), probably due to the TEA^•^ radical undergoing side reactions such as decomposition or dimerization in alkaline environments. Such a pH‐sensitive ECL response held great potential for monitoring dynamic change of pH in localized cellular environments or tracking pH fluctuations during specific enzymatic reactions.

### 2.5 ECL Mechanism of TEA‐Pdots

**FIGURE 4:**
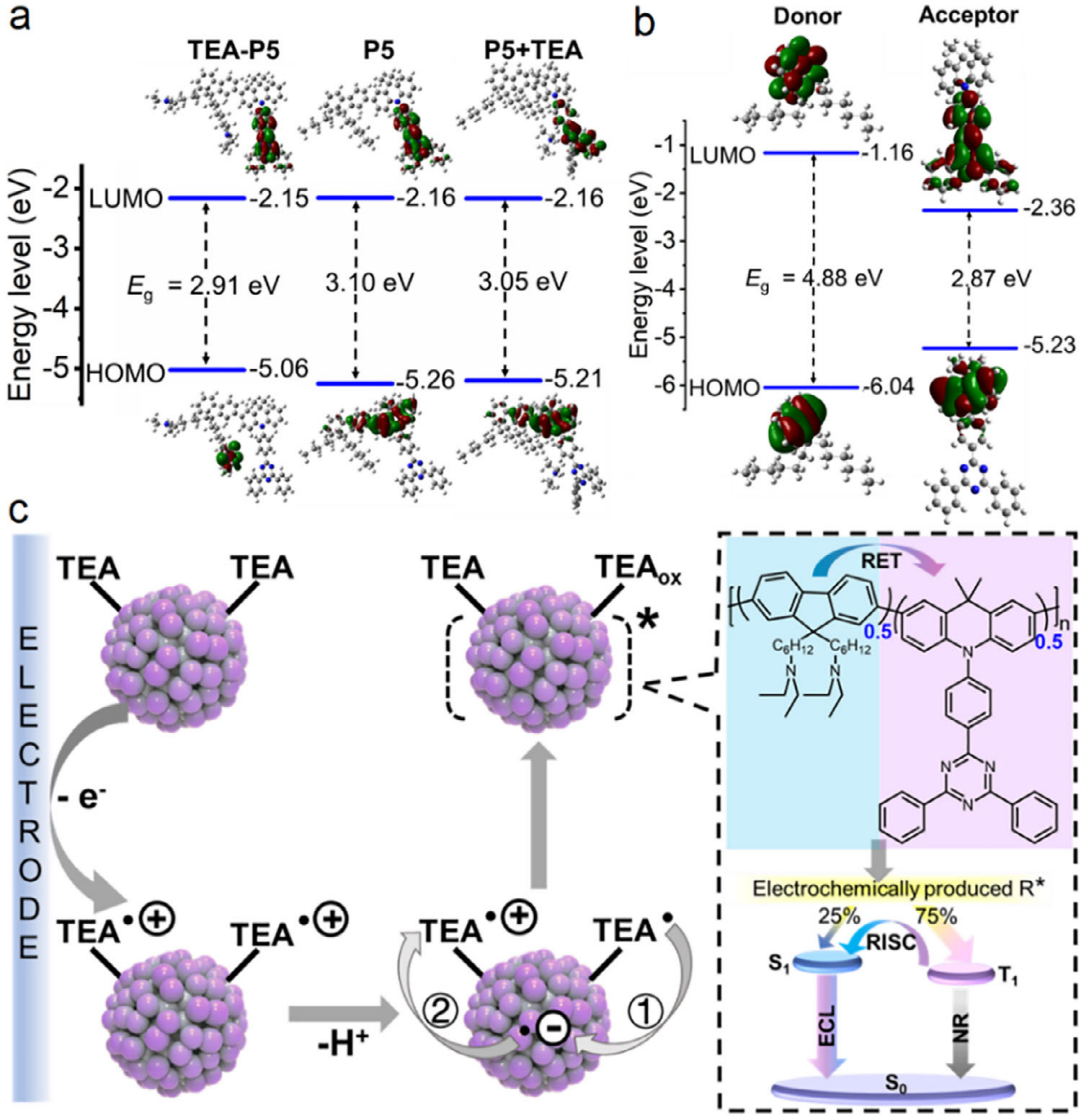
(a, b) Calculated spatial distributions, and orbital energy levels of HOMO and LUMO in TEA‐P5, P5 and TEA‐mixed P5 (a), and donor and acceptor (b) based on the optimized ground‐state geometric structures. (c) Schematic diagram of ECL mechanism of TEA‐Pdots5.

To reveal the ECL mechanism of TEA‐Pdots5, the highest occupied and the lowest unoccupied molecular orbital (HOMO and LUMO) energy levels of TEA‐P5, P5, and TEA mixed P5 (P5+TEA) were calculated with density functional theory (Figure 4a). The LUMO of TEA‐P5 was localized on the triazine moiety and adjacent benzene rings, while its HOMO primarily resided on one of the tertiary amine groups, indicating the preferential oxidation of tertiary amine in TEA‐P5. In contrast, both the HOMOs of P5 and P5+TEA were distributed on acridine and fluorine, indicating the negligible orbital perturbation by exogenous TEA. The orbital analysis of donor and acceptor showed that the emission energy of donor exceeded the absorption energy of acceptor, and the orbital alignment was favorable for energy transfer (Figure 4b). Consequently, efficient RET via dipole‐dipole coupling was thermodynamically and kinetically feasible, while charge transfer was energetically unfavorable [36].

From the experimental and theoretical calculation results, the detailed ECL mechanism of TEA‐Pdots5 was illustrated in Figure 4c. First, the TEA^•+^‐Pdots5 was produced via the oxidization of tertiary amine groups and then deprotonation. Due to the more positive reduction potential of Pdots5 (−1.45 V, Figure S9) than that of TEA^•^ (−1.7 V) [37], one electron within TEA^•^ could be intramolecularly transfered to Pdots5 to produce TEA‐Pdots5^•−^. Considering each luminescent unit in TEA‐Pdots5 contained two TEA fragments, the electron within TEA‐Pdots5^•−^ could be further transfered to another TEA^•+^, producing the excited state (TEA‐Pdots5^*^), followed by the transfer of excited energy from fluorene to DMAC‐TRZ via RET. Finally, the triplet excitons of DMAC‐TRZ could be transfered to the singlet state via RISC [38], enabling TEA‐Pdots5* to efficiently harvest excitons for ECL emission.

To highlight the superiorities of TEA‐Pdots5, the monomer of TEA‐P5 (**M‐4**) and the polymer without tertiary amine modification (**P5**) were synthesized (Scheme S1) to prepare the corresponding TEA‐Dots and Pdots5 (Figure S10a). Compared to TEA‐Dots, TEA‐Pdots5 exhibited a 20‐nm red‐shift of FL emission peak at 525 nm with 5‐fold enhanced intensity (Figure S10b), attributable to its conjugated structure [20]. This conjugated structure accelerated electron transfer through enhanced *p*‐orbital overlap, resulting in a 159‐fold stronger ECL emission than that of TEA‐Dots (Figure S10c). Notably, both TEA‐Dots and TEA‐Pdots5 displayed ECL peaks at +0.96 V, representing a negative shift of 0.30 V relative to Pdots5 (Figure S10d). Such a low ECL potential (Table S3) could minimize side reactions during anodic ECL process and reduce cellular damage caused by electrochemical signal stimulation during ECL imaging [39], which was favorable for ECL bioanalysis. In contrast, in the presence of 10 mM TEA, Pdots5 showed a weak ECL emission at +1.30 V with 58‐fold lower intensity than that of TEA‐Pdots5 (Figure S10d), highlighting the critical role of intramolecular electron transfer pathways in TEA‐Pdots5 vs. inefficient intermolecular mechanism of Pdots5.

### 2.6 ECL Efficiencies of TEA‐Pdots

TEA‐Pdots1∼TEA‐Pdots5 exhibited similar ECL emissions at around 527 nm (Figure S11), closely matching their FL wavelengths (Figure 3a), indicating the same excited states shared in FL and ECL processes [40]. Additionally, TEA‐Pdots1 and TEA‐Pdots2 also exhibited weak ECL emissions related to the donor in the range of 400–450 nm (Figure S12a,b), consistent with their FL emission peaks, suggesting the sufficient RET process in TEA‐Pdots.

According to the synthesis procedure, the number of luminescence units per TEA‐Pdots5 was estimated to be 154. Given that each luminescent unit contains two amine groups, it follows that a single TEA‐Pdot5 contains a total of 308 amine groups (see Supporting Information). Thus, the concentration of tertiary amine in 100 µg mL^−1^ TEA‐Pdots was significantly higher than 200 µm. The *Φ_ECL_* of TEA‐Pdots modified on GCE was measured under the conditions shown in Figures S12 and S13, which revealed a progressive *Φ_ECL_* enhancement from TEA‐Pdots1 to TEA‐Pdots5 (Table S4). Significantly, TEA‐Pdots5 achieved a record *Φ_ECL_* of 92.6% against the standard system (1 mm [Ru(bpy)_3_]^2+^/10 mm TPrA), which was 5.42‐fold higher than that of equimolar 100 µm [Ru(bpy)_3_]^2+^/200 µm TEA and outperformed state‐of‐the‐art TADF Pdots [32], DMAC‐TRZ nanoparticles [38], and PFBT‐derived TEA‐Pdots (Table S5) [20]. This breakthrough originated from three synergistic mechanisms: (i) fluorene enables highly efficient luminescence as the energy donor, while DMAC‐TRZ maximizes exciton utilization as the acceptor; (ii) the coreactant‐embedded conjugated superstructure in TEA‐Pdots5 facilitated dual intramolecular electron transfer, effectively generating stabilized excited states; (iii) the ARET mechanism enhances the energy transfer from the excited state to TADF emitters with near‐unity exciton harvesting. The concerted integration of highly efficient luminescence and efficient exciton utilization along with the relatively low anodic ECL potential thereby culminated in unprecedented ECL amplification of TEA‐Pdots, manifesting profound promise in single‐cell ECL microimaging.

### 2.7 ECL Bioimaging

**FIGURE 5:**
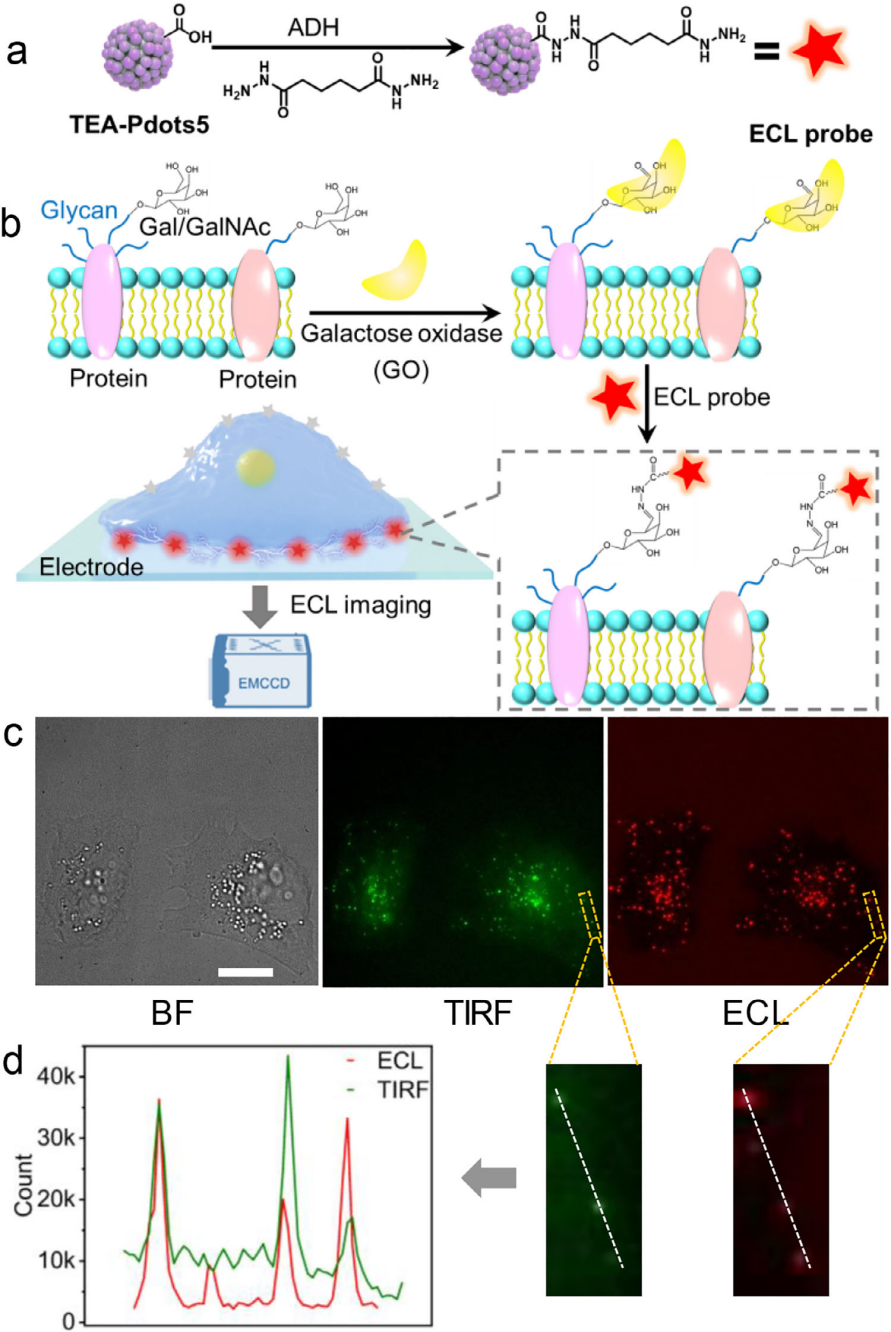
(a, b) Schematic diagrams of ECL probe preparation (a) and ECL imaging of terminal Gal/GalNAc on cell surface (b,c) BF, TIRF, and ECL images. Scale bar: 10 µm. (d) ECL and TIRF profiles along the white dashed lines of Gal/GalNAc on HeLa cells.

The synthesized TEA‐Pdots5 not only possessed the advantages of small particle size, low ECL peak potential, high *Φ_ECL_*, and coreactant‐free operation, but also exhibited minimal cytotoxicity compared to the significantly higher toxicity of exogenous TEA (Figure S14), making them particularly suitable for live‐cell ECL imaging. As proof‐of‐concept, the terminal galactose/N‐acetylgalactos‐amine (Gal/GalNAc) on cell surface, which plays a critical role in cellular recognition, signal transduction, immune responses and tumorigenesis [41, 42], was chosen as the imaging target. First, the surface‐exposed carboxyl groups of TEA‐Pdots5 were coupled with ADH to yield hydrazide‐functionalized TEA‐Pdots5 as an ECL probe (Figure 5a), which could specifically link with NHS‐activated cyanine5 (NHS‐Cy5) (Figure S15). The DLS characterization confirmed its monodispersed in working buffer (Figure S16), eliminating the possibility of pre‐existing aggregates before cellular application. Subsequently, the C6‐hydroxy group of terminal Gal/GalNAc on cell surface was enzymatically oxidized by galactose oxidase (GO) to generate the corresponding aldehyde [43], followed by hydrazone ligation with the ECL probe for ECL imaging (Figure 5b).

The ECL imaging was performed using homemade ultrathin indium tin oxide (ITO) slide as the working electrode [2]. The TEA‐Pdots5 modified ITO electrode exhibited an ECL emission at +1.02 V with onset at +0.81 V (Figure S17), showing a slight positive shift compared to the potential observed on GCE. In comparison with total internal reflection fluorescence (TIRF) imaging, the proposed ECL imaging detection showed a higher abundance of terminal Gal/GalNAc on HeLa cells due to the strong ECL emission of TEA‐Pdots5 (Figure 5c), leading to higher target sensitivity with superior signal‐to‐background ratio (Figure 5d). To be noted, due to the natural enrichment of Gal/GalNAc in specific membrane microdomains such as lipid rafts [41, 44, 45], the target Gal/GalNAc is not uniformly distributed on the cell surface, resulting in a non‐uniform probe binding pattern. Moreover, the ECL and TIRF profiles were partially mismatched (Figure 5d; Figure S18) due to the fundamentally different imaging mechanisms between ECL and TIRF. Regions with high TIRF but low ECL signal likely contained the probes trapped in locations with poor electrochemical communication with the electrode. Conversely, regions with strong ECL emission might result from optimal interface for electron transfer and coreactant generation. This unique ECL feature provided complementary spatial information about the electrochemical microenvironment at the cell‐electrode interface.

The proposed ECL imaging method underscored its advantages in membrane glycan profiling on living cells by eliminating the needs for externally‐added coreactants and excitation light interference, while providing subcellular spatial resolution, thus demonstrating its utility for low‐abundance glycan analysis.

## 3 Conclusion

In summary, this study pioneers an ARET mechanism to break through the key limitation in *Φ*_ECL_ of ECL emitters by integrating an AIE‐active fluorene donor, a TADF acceptor, and tertiary amine coreactant in Pdots. This mechanism leads to a marked and continuous acceptor emission amplification of the polymers in THF/water mixture with increasing water fraction via boosting the quantum yield. Based on the aggregation amplified ECL emission of the polymers, the proposed TEA‐Pdots demonstrate the ARET effect and a relatively low oxidation potential due to the dual intramolecular electron transfer from TEA^•^ to Pdots and then TEA^•+^ for producing the excited state, which achieves an *Φ_ECL_* record of 92.6%, representing 5.42‐fold higher than that of equimolar [Ru(bpy)_3_]^2^^+^ and significantly outperforming the existing organic nanomaterials. This unprecedented ECL performance arises from the synergistic interplay of (i) highly emissive AIE donor, (ii) efficient exciton harvesting via TADF in the acceptor, (iii) facilitated dual intramolecular electron transfer enabled by the embedded tertiary amines, and (iv) ARET effect within the nanostructure. Benefiting from the low ECL potential, high *Φ_ECL_*, and minimal cytotoxicity, TEA‐Pdots enable sensitive ECL imaging of terminal Gal/GalNAc on HeLa cells, demonstrating the practical utility. Overall, the proposed ARET strategy achieves a new paradigm in designing high‐performance ECL emitters for advanced bioanalysis and bioimaging.

## 4 Experimental Section

### 4.1 Synthesis of Model Monomer and Conjugated Polymers

The synthesis routes of monomer and polymers were outlined in Scheme S1. The monomer was one unit of the TEA‐P5, marked as M‐4. The polymers included those without tertiary amine groups (P1∼P5) and conjugated with diethylamine (TEA‐P1∼TEA‐P5).

### 4.2 Preparation of Pdots1∼Pdots5, TEA‐Pdots1∼TEA‐Pdots5 and Dots

Taking the preparation of Pdots1 as an example, after 2.5 mL THF solution containing 50 µg·mL^−1^ P1 polymer and 10 µg·mL^−1^ PSMA were ultrasonically degassed at RT for 20 min, the mixture was rapidly injected into 10 mL water with sonication for another 30 s. Subsequently, THF was evaporated using a rotary evaporator, and the resulting solution was filtrated via a 0.22 µM poly(ether sulfones) syringe filter to obtain carboxyl Pdots1 dispersion. Pdots2∼Pdots5, TEA‐Pdots1∼TEA‐Pdots5, and Dots were prepared following the same procedure by substituting P1 with P2∼P5, TEA‐P1∼TEA‐P5, and M‐4, respectively.

### 4.3 Preparation of ECL Probes

For the preparation of ECL probes, 600 µL TEA‐Pdots5 dispersion was mixed with PEG (5% w/v, 12 µL) and HEPES (1 m, 12 µL), and the pH was adjusted to 7.4 with 0.1 m NaOH. Then, freshly prepared EDC (10 mg·mL^−1^, 24 µL) and ADH (400 mm, 500 µL) were added into the mixture and stirred gently for 2 h. The obtained mixture was ultra‐filtrated for six times to remove unreacted ADH and then diluted to an appropriate concentration for subsequent experiments.

### 4.4 Cell Treatment for ECL Imaging

HeLa cells were cultured in DMEM medium supplemented with 10% FBS and 1% penicillin‐streptomycin at 37°C in a humidified atmosphere containing 5% CO_2_. For the detection of terminal Gal/GalNAc on HeLa cells, the cells were washed with PBS (1×) for thrice and blocked with PBS containing 10% goat serum at 4°C for 1 h to reduce non‐specific adsorptions, followed by incubating with PBS containing 0.01 mg·mL^−1^ GO at 37°C for 30 min. After washing with PBS for thrice, the cells were treated with PBS containing 100 µg mL^−1^ TEA‐Pdots5‐ADH, 10 mm aniline, and 5% FBS at 37°C for 30 min. After washing gently with PBS for thrice, the cells were digested with 0.1% trypsin for 40 s and stopped by adding culture medium. After centrifugation, the cells were suspended in 1 mL of DMEM medium to seed on a homemade ultrathin ITO electrode at 37°C for 6 h [2] and then perform ECL imaging in PBS (1×).

### 4.5 TIRF and ECL Imaging of Cells

The photoluminescence and ECL images of HeLa cells were taken using a TIRF microscope (DMI8‐TIRF AM, Leica, Germany) equipped with an ultrasensitive EMCCD camera (iXon Ultra 897, Andor, UK) operating at single‐photon sensitivity. The system was controlled by ANDOR SOLIS software and cooled to −95°C. Photons were collected by an oil immersion objective with a high numerical aperture (HCX PL APO 100×/1.47). For TIRF imaging, a 488‐nm laser served as the excitation source. The fluorescence filter cube consisted of a 500‐nm dichroic mirror and a 520‐nm long‐pass emission filter, matched to the emission spectrum of TEA‐Pdots5. For ECL imaging, the ECL signal was excited by a CHI‐660D electrochemical workstation with the cell‐seeded ITO slip as working, a Pt wire as the counter, and an Ag/AgCl wire as the reference electrodes. The ECL images were recorded at an exposure time of 40 s during cyclic voltammetric scans (0 to +1.0 V, 0.5 V s^−^^1^), accumulating the signal over 10 cycles to minimize cycle‐to‐cycle variability.

### 4.6 ECL Spectra

CHI 630D electrochemical workstation (CHI instruments Inc., China) conjugating with a GCFG‐B ECL analyzer (Shandong Guochen Biotech Co., Ltd., China) was used to record the ECL spectra by using Ag/AgCl as reference and Pt wire as counter electrodes. The working electrode was first modified with an aqueous solution of 1 mm [Ru(bpy)_3_]^2^^+^, 100 µm [Ru(bpy)_3_]^2^^+^, or TEA‐Pdots dispersion. After allowing the solution to dry, 20 µL of 0.1 wt.% ethanolic Nafion solution was drop‐cast on the electrode surface. The ECL spectra was recorded concurrently with cyclic potential scans from +0.0 to +1.5 V. The ECL spectrum of 1 mm [Ru(bpy)_3_]^2+^ modified electrodes was recorded in 0.1 m PBS (pH 7.4) containing 0.1 m KNO_3_ and 10 mm TPrA. The ECL spectrum of 100 µm [Ru(bpy)_3_]^2+^ modified electrodes was recorded in 0.1 m PBS (pH 7.4) containing 0.1 m KNO_3_ and 200 µm TEA. The ECL spectrum of TEA‐Pdots modified electrode was recorded in 0.1 m PBS (pH 7.4) containing 0.1 m KNO_3_. The relative *Φ_ECL_* was calculated using the equation:


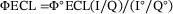

where *Φ_ECL_* and *Φ*°_ECL_, *I* and *I*°, *Q* and *Q*° stand for the ECL efficiency, intensity integral (integrating ECL spectrum vs. wavelength), consumed charge (integrating CV curve vs. time) of TEA‐Pdots and [Ru(bpy)_3_]^2+^ systems, respectively [46]. In this work, 1 mm [Ru(bpy)_3_]^2+^/10 mm TPrA was used as the standard with the *Φ*°*_ECL_* value of 1.

### 4.7 Statistical Analysis

All statistical data in this work were presented as the mean ± standard error of the mean. Data were normalized by dividing each value by the mean of its respective dataset. Error bars represent the standard deviation derived from three independent replicates. The line plots, bar charts, and scatter plots with connecting lines were generated using Origin software (version 9.0). Microscopy images were analyzed using ImageJ Fiji software.

## Funding

This work was financially supported by the National Natural Science Foundation of China (21890741).

## Conflicts of Interest

The authors declare no conflicts of interest.

## Supporting information




**Supporting File**: advs74008‐sup‐0001‐SuppMat.docx.

## Data Availability

The data that support the findings of this study are available from the corresponding author upon reasonable request.
