# Supplementary material for: Aggregation‐Induced Resonance Energy Transfer in Polymer Dots to Boost Electrochemiluminescence Performance for Bioimaging of Glycan on Cells
Source: Adv Sci (Weinh). 2026 Jan 22;13(19):e24265. doi: 10.1002/advs.202524265 (PMC13045401; doi:10.1002/advs.202524265)
Supplement: Supplementary file 1 — Supporting File: advs74008‐sup‐0001‐SuppMat.docx. [file ADVS-13-e24265-s001.docx]

**Supporting Information**

**Aggregation-Induced Resonance Energy Transfer in Polymer Dots to Boost Electrochemiluminescence** **Performance for Bioimaging of Glycan on Cell**

Chao Wang,^[a, b]^ Mengjiao Li,^[a]^ Yiran Li,^[a]^ Xiangfu Hu,^[a]^ Chunlan Li,^[c]^ Ningning Wang,^[b]^ and Huangxian Ju*^[a]^

^[a]^ State Key Laboratory of Analytical Chemistry for Life Science, School of Chemistry and Chemical Engineering, Nanjing University, Nanjing 210023, China

^[b]^ School of Pharmacy, Binzhou Medical University, Yantai, 264003, China

^[c]^ Henan Key Laboratory of Biomolecular Recognition and Sensing, Henan Joint International Research Laboratory of Chemo/Biosensing and Early Diagnosis of Major Diseases, College of Chemistry and Chemical Engineering, Shangqiu Normal University, Shangqiu 476000, China

*Corresponding author. Email: hxju@nju.edu.cn

**Experimental Section**

**Materials and Reagents.** 10-(4-(4, 6-Diphenyl-1, 3, 5-triazin-2-yl)phenyl)-9, 9-dimethyl-9, 10-dihydroacridine (DMAC-TRZ) was synthesized according to a previous work.^[S1]^ Potassium tert-butoxide (KTB), tetrakis(triphenylphosphine)palladium (Pd(PPh_3_)_4_), 2-(9,9-bis(6-bromohexyl)-9H-fluoren-2-yl)-4,4,5,5-tetramethyl-1,3,2-boronic acid ester (**M-2**), 2,7-dibromo-9,9-bis(6-bromohexyl)fluorene (**M-6**), 2,2’-(9,9-bis(6-bromohexyl)-9H-fluorene-2,7-diyl)bis(4,4,5,5-tetramethyl-1,3,2-dioxaborolane) (**M-7**), and 1-bromopyrrolidine-2,5-dione (NBS) were obtained from Bide Pharmaceutical Technology Co., Ltd. (Shanghai, China). Diethylamine, tripropylamine (TPrA), and triethylamine (TEA) were obtained from J&K Chemical Ltd. (Beijing, China). K_2_CO_3_ was purchased from Nanjing Chemical Reagent Co., Ltd. (Nanjing, China). Poly(styrene-co-maleic anhydride) (PSMA, average Mn: 1700), 4-(2-hydroxyethyl)-1-piperazineethanesulfonic acid (HEPES), polyethylene glycol (PEG, average Mw: 3350), *N*-(3-dimethylaminopropyl)-*N*’-ethylcarbodiimide hydrochloride (EDC), sulfo-cyanine 5-*N*-hydroxysuccinimide ester (Cy5-NHS), and adipic dihydrazide (ADH) were purchased from Sigma-Aldrich Co., Ltd. (Shanghai, China). Galactose oxidase (GO) was purchased from Sangon Biotech Inc. (Shanghai, China). Aniline, toluene, tetrahydrofuran (THF), and *N*, *N*-dimethylformamide (DMF) were purchased from Macklin Biochemical Co., Ltd. (Shanghai, China). Fetal bovine serum (FBS), phosphate buffered saline (PBS, pH 7.4), HeLa cells, Dulbecco’s Modified Eagle’s Medium (DMEM) cell culture media, and trypsin were supplied by KeyGen Biotech Co., Ltd. (Nanjing, China). All aqueous solutions were prepared using ultrapure water (≥ 18 MΩ, Milli-Q, Millipore).

**Apparatus.** The ^1^H nuclear magnetic resonance (NMR) spectra were recorded on a Bruker AvanceIII 400 MHz NMR spectrometer using CDCl_3_ as solvent and tetramethylsilane as internal standard. Gel permeation chromatographic (GPC) analysis was performed on a Waters 410 system with polystyrene as the standard and THF as the eluent. Transmission electron microscopic (TEM) images were obtained on a JEM-2100 transmission electron microscope (JEOL, Japan). UV-vis absorption spectra were recorded on a UV-3600 spectrophotometer (Shimadzu, Japan). Fluorescence (FL) spectra and fluorescence decay curves were measured using an FLS-980 fluorescence spectrophotometer (Edinburgh, U.K.). The FL excitation wavelength (*λ*_ex_) for TEA-Ps and TEA-Pdots, DMAC-TRZ, and Cy5 were 300 nm, 365 nm, and 620 nm, respectively. Phosphorescence spectrum was collected at 77 K with a time delay of 10 ms between the pulse excitation and the collection of emission spectrum. Dynamic light scattering (DLS) and Zeta potential measurements were conducted using a 90 Plus/BI-MAS equipment (Brookhaven Instruments Co., USA). X-ray photoelectron spectroscopy (XPS) data were acquired using a PHI5000 X-ray photoelectron spectrometer (Ulvac-Phi, Japan). Electrochemiluminescence (ECL) spectra were obtained on a GCFG-B ECL analyzer (Shandong Guochen Biotech Co., Ltd., China). Voltammetric experiments were performed on a CHI 630D electrochemical workstation (CH Instruments Inc., China), while ECL measurements were conducted with a self-made cell on a MPI-EII ECL analyzer (Xi’an Remex, China) with a three-electrode system, including a modified glassy carbon as working electrode (GCE, diameter of 5 mm), a Pt counter electrode, and a Ag/AgCl (saturated KCl) reference electrode. Unless otherwise specified, the voltage of photomultiplier tube (PMT) was set at 400 V, and the scan rate was set at 0.1 V s^-1^. The theoretical calculations of the highest occupied molecular orbital (HOMO) and the lowest unoccupied molecular orbital (LUMO) were carried out with Gaussian 16 A.03 software.^[S2]^ The monomer of TEA-P5 and its similar molecule were optimized using dispersion-corrected density functional theory (DFT-D3) at B3LYP-D3/def2-TZVP level using the ORCA program. The excited electronic structure was calculated using time-dependent density functional theory (TD-DFT) at the M06-D3/def2-TZVP level, providing both excited energies and oscillator strengths. Natural transition orbitals (NTOs) and molecular orbital transition contributions were obtained using the Multiwfn program.^[S3]^

**Synthesis of M-1 and M-5.** 10-Dihydroacridine (**M-1**), 2-bromo-10-(4-(4,6-diphenyl-1,3,5-triazin-2-yl)phenyl)-9,9-dimethyl-9, and 2,7-dibromo-10-(4-(4,6-diphenyl-1,3,5-triazin-2-yl)phenyl)-9,9-dimethyl-9,10-dihydroacridine (**M-5**) were prepared according to a previous work.^[S4]^ After 5 mL DMF solution containing DMAC-TRZ (0.40 g, 0.77 mmol) and NBS (0.14 g, 0.80 mmol) was stirred under an argon atmosphere in a 25 mL Schlenk tube at 115 °C for 48 h, it was quenched by methanol. The product was extracted with dichloromethane and purified by silica gel column chromatography with petroleum ether/dichloromethane as eluent to obtain 0.3768 g **M-1** (yield 82%). For the synthesis of **M-5**, double amount of NBS was used to obtain 0.4363 g **M-5** (yield 84%). The ^1^H NMR of **M-1** (400 MHz, CDCl_3_, Appendix S1): *δ =* 9.03 (d, *J* = 8.4 Hz, 2H), 8.82 (d, *J* = 7.8 Hz, 4H), 7.70-7.43(m, 10 H), 7.07(d, *J* = 8.8 Hz, 1H), 6.98 (dd, *J* = 12.5, 6.2 Hz, 2H), 6.37(dd, *J* = 7.3, 2.0 Hz, 1H), 6.24(dd, *J* = 8.8, 2.4 Hz, 1H), 1.71(t, *J* = 8.7 Hz, 3H). The ^1^H NMR of **M-5** (400 MHz, CDCl_3_, Appendix S2) *δ =* 9.03 (d, *J* = 8.3 Hz, 2H), 8.81 (d, *J* = 6.9 Hz, 4H), 7.68-7.48(m, 10 H), 7.08 (dd, *J* = 8.8, 2.2 Hz, 2H), 6.24 (d, *J* = 8.8 Hz, 2H), 1.69 (s, 6H).


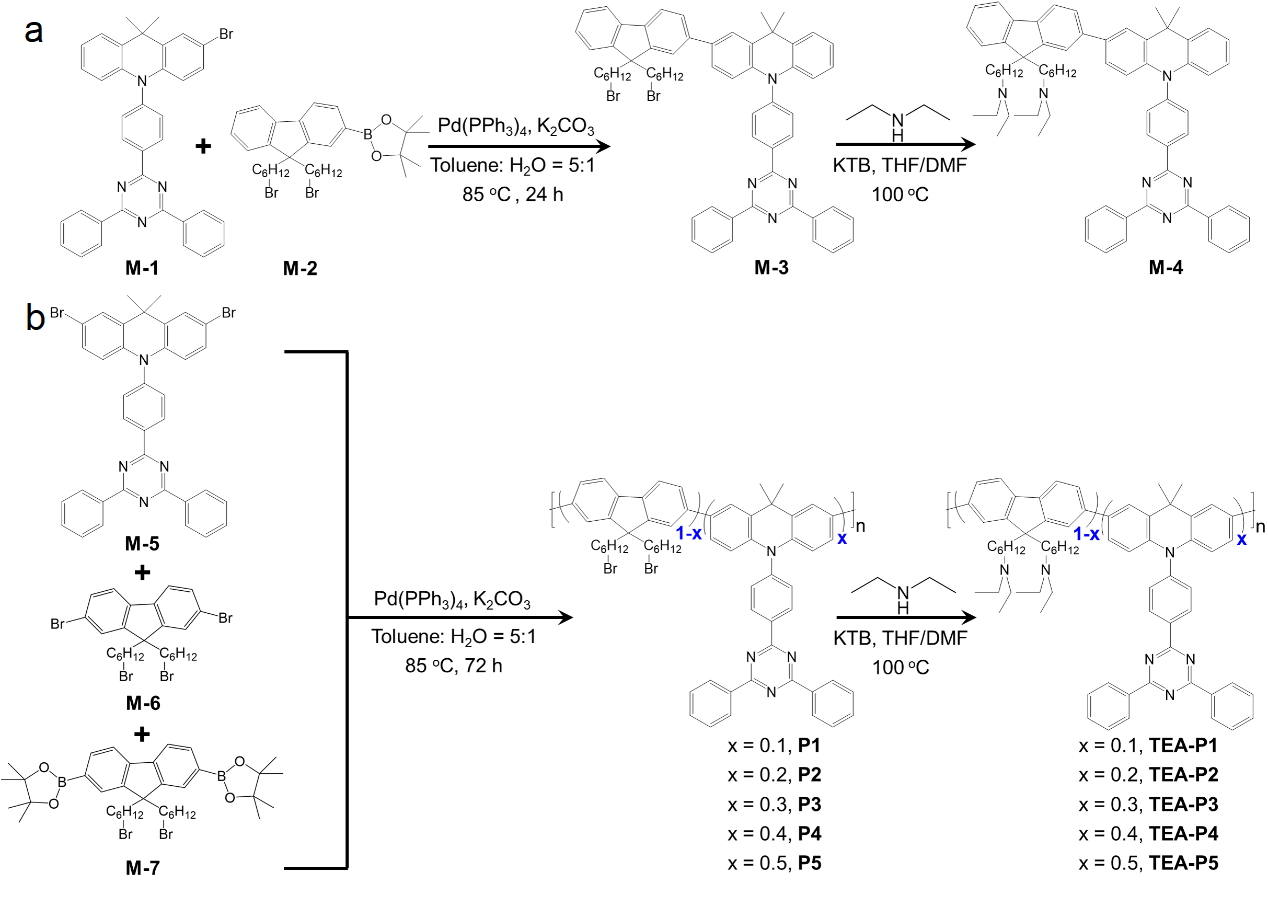


**Scheme S1.** Synthetic routes of (a) **M-3**, and **M-4**, and (b) P1~P5, and TEA-P1~TEA-P5.

**Synthesis of M-3.** After 5 mL toluene solution containing **M-1** (0.060 g, 0.10 mmol), **M-2** (0.062 g, 0.10 mmol), Pd(PPh_3_)_4_ (0.0060 g, 0.005 mmol) and K_2_CO_3_ (0.11 g, 0.8 mmol) and 1 mL H_2_O were mixed to stir in a 25 mL Schlenk tube at 85 °C for 24 h under an argon atmosphere, the product was extracted with dichloromethane and purified by silica gel column chromatography with petroleum ether/dichloromethane as eluent to obtain 0.0817 g **M-3** (yield 81%). The ^1^H NMR of **M-3** (400 MHz, CDCl_3_, Appendix S3): *δ =* 9.06 (d, *J* = 8.1 Hz, 2H), 8.83 (d, *J* =6.6 Hz, 4H), 7.78-7.52 (m, 14H), 7.36-7.29 (m, 4H), 7.01(d, *J* =7.0 Hz, 2H), 6.45 (dd, *J* =27.6, 8.0 Hz, 2H), 3.38 (t, *J* =6.8 Hz, 1H), 3.25 (t, *J* = 6.8 Hz, 3H), 2.02-1.96 (m, 4H), 1.83 (s, 6H), 1.63 (dd, *J* = 14.5, 7.1 Hz, 4H), 1.18 (d, *J* = 7.4 Hz, 4H), 1.10-1.05 (m, 4H), 0.68 (dd, *J* = 15.3, 7.4 Hz, 4H).

**Synthesis of M-4.** After **M-3** (50 mg), diethylamine (0.5850 g, 0.008 mol) and KTB (0.2244 g, 0.002 mol) were dissolved in the mixture of 8 mL THF and 2 mL DMF to stir in a 25 mL Schlenk tube at 100 °C for 96 h under an argon atmosphere, the product was extracted with dichloromethane and purified by silica gel column chromatography with petroleum ether/dichloromethane as eluent to obtain 0.0386 g **M-4** (yield 78%). The ^1^H NMR of **M-4** (400 MHz, CDCl_3_, Appendix S4): *δ =* 9.06 (s, 2H), 8.83 (d, *J* = 6.5 Hz, 4H), 7.78-7.42 (m, 14H), 7.37-7.27 (m, 4H), 7.00 (s, 2H), 6.44 (s, 2H), 2.03-1.98 (m, 4H), 1.86-1.75 (m, 10H), 1.69-1.58 (m, 4H), 1.31-1.12 (m, 24H), 0.88 (t, *J* = 6.8 Hz, 4H), 0.70 (s, 4H).

**Synthesis of Polymers P1~P5.** For the synthesis of **P1**, **M-5** (0.01812 g, 0.0269 mmol), **M-6** (0.070 g, 0.1075 mmol), **M-7** (0.1000 g, 0.1344 mmol), Pd(PPh_3_)_4_ (0.0156 g, 0.01344 mmol), and K_2_CO_3_ (0.1857 g, 1.3440 mmol) were dissolved in the mixture of 5 mL toluene and 1 mL H_2_O, and stirred in a 25 mL Schlenk flask at 85 °C for 72 h under an argon atmosphere. The resulting product was extracted with dichloromethane and purified by precipitation in methanol as light yellow solid (0.0968 g, yield: 73%). The ^1^H NMR of P1 (400 MHz, CDCl_3_, Appendix S5): *δ =* 9.06 (s, 0.2H), 8.82 (s, 0.4H), 7.87-7.45 (m, 6.4H), 7.00 (s, 0.2H), 6.83 (s, 0.2H), 3.36 (t, *J* = 44.7 Hz, 3.6H), 2.14 (s, 3.6H), 1.79 (s, 0.6H), 1.68 (s, 3.6H), 1.21 (d, *J* = 32.3 Hz, 7.2H), 0.83 (s, 3.6H). GPC data: M_n_ = 16831, M_w_ = 23582, PDI = 1.40.

**P2** was synthesized following the same procedure as **P1**, using **M-5** (0.03625 g, 0.0537 mmol), **M-6** (0.05241 g, 0.0806 mmol) and **M-7** (0.1000 g, 0.1344 mmol), yielding 0.0918 g of **P2** (yield 69%). The ^1^H NMR of **P2** (400 MHz, CDCl_3_, Appendix S6): *δ =* 9.04 (s, 0.4H), 8.83 (s, 0.8H), 7.80-7.41 (m, 6.8H), 7.00 (s, 0.4H), 6.81 (s, 0.4H), 3.39 (d, *J* = 91.5 Hz, 3.2H), 2.13 (s, 3.2H), 1.79 (s, 1.2H), 1.68 (s, 3.2H), 1.29-1.10 (m, 6.4H), 0.82 (s, 3.2H). GPC data: M_n_ = 15695, M_w_ = 24823, PDI = 1.58.

**P3** was synthesized following the same procedure as **P1**, using **M-5** (0.05437 g, 0.0806 mmol), **M-6** (0.03494 g, 0.0537 mmol) and **M-7** (0.1000 g, 0.1344 mmol), yielding 0.0879 g of **P3** (yield 66%). The ^1^H NMR of **P3** (400 MHz, CDCl_3_, Appendix S7): *δ =* 9.04 (s, 0.6H), 8.79 (d, *J* = 20.4 Hz, 1.2H),7.89-7.43 (m, 7.2H), 7.00 (s, 0.6H), 6.81 (s, 0.6H), 3.28 (s, 2.8H), 1.88 (dd, *J* = 125.4, 56.7 Hz, 7.4H), 1.20 (m, 5.6H), 0.81 (s, 2.8H). GPC data: M_n_ = 15063, M_w_ = 24467, PDI = 1.62.

**P4** was synthesized following the same procedure as P1, using **M-5** (0.07248 g, 0.1075 mmol), **M-6** (0.01747 g, 0.0269 mmol) and **M-7** (0.1000 g, 0.1344 mmol), yielding 0.0806 g of **P4** (yield 60%). The ^1^H NMR of **P4** (400 MHz, CDCl_3_, Appendix S8): *δ =* 9.06 (s, 0.8H), 8.82 (s, 1.6H), 7.64 (t, *J* = 57.0 Hz, 7.8H), 7.02 (s, 0.8H), 6.80 (s, 0.8H), 3.39 (d, *J* = 100.3 Hz, 2.4H), 1.98 (dd, *J* = 81.4, 47.9 Hz, 7.2H), 1.24 (m, 4.8H), 0.82 (s, 2.4H). GPC data: M_n_ = 13608, M_w_ = 22724, PDI = 1.67.

**P5** was synthesized following the same procedure as **P1**, using **M-5** (0.0906 g, 0.1344 mmol) and **M-7** (0.1000 g, 0.1344 mmol), yielding 0.0762 g of P5 (yield 56%). The ^1^H NMR of **P5** (400 MHz, CDCl_3_, Appendix S9): *δ =* 8.98 (s, 1H), 8.74 (s, 2H), 7.83-7.32 (m, 8H), 6.99 (s, 1H), 6.74 (s, 1H), 3.29 (d, *J* = 91.9 Hz, 2H), 2.16-1.61 (m, 6.8H), 1.09 (m, 3H), 0.84-0.52 (s, 3H). GPC data: M_n_ = 12984, M_w_ = 22869, PDI = 1.76.

**Synthesis of Polymers TEA-P1~TEA-P5.** For the synthesis of TEA-P1, **P1** (50 mg), diethylamine (0.5850 g, 0.008 mol) and KTB (0.2244 g, 0.002 mol) were added into a 25 mL Schlenk tube containing 8 mL THF and 2 mL DMF and stirred at 100 °C for 96 h under an argon atmosphere. The resulting product was extracted with dichloromethane and purified by precipitation in methanol to obtain 0.7512 g TEA-P1 (yield 86%). The TEA-P2~TEA-P5 were synthesized using the same procedure with **P2**~**P5**, respectively.

**TEA-P1**: ^1^H NMR (400 MHz, CDCl_3_, Appendix S10) *δ =* 9.00 (s, 0.2H), 8.75 (s, 0.4H), 7.93-7.26 (m, 6.4H), 6.89 (s, 0.2H), 6.74 (s, 0.2H), 2.2-1.6 (m, 11.4H), 1.4-0.6 (m, 28.8H).

**TEA-P2**: ^1^H NMR (400 MHz, CDCl_3_, Appendix S11) δ = 9.08 (s, 0.4H), 8.83 (s, 0.8H), 7.8-7.4 (m, 6.8H), 7.00 (s, 0.4H), 6.82 (s, 0.4H), 2.2-1.7 (m, 10.8H), 1.4-0.7 (m, 25.6H).

**TEA-P3**: ^1^H NMR (400 MHz, CDCl_3_, Appendix S12) *δ =* 9.05 (s, 0.6H), 8.83 (s,1.2H), 7.8-7.4 (m, 7.2H), 7.0 (s, 0.6H), 6.81 (s, 0.6H), 2.2-1.6 (m, 10.2H), 1.3-0.6 (m, 22.4H).

**TEA-P4**: ^1^H NMR (400 MHz, CDCl_3_, Appendix S13) *δ =* 8.98 (s, 0.8H), 8.74 (s, 1.6H), 7.8-7.4 (m, 7.6H), 6.93 (s, 0.8H), 6.72 (s, 0.8H), 2.2-1.6 (m, 9.6H), 1.2-0.6 (m, 19.2 H).

**TEA-P5**: ^1^H NMR (400 MHz, CDCl_3_, Appendix S14) *δ =* 9.07 (s, 1H), 8.83 (s, 2H), 7.8-7.4 (m, 8H), 7.00 (s, 1H), 6.79 (s, 1H), 2.2-1.6 (m, 9H), 1.4-0.7 (m, 16H).

**Estimation of the Number of Luminescent Units and Coreactant Per TEA-Pdot5.** The number of luminescent units per TEA-Pdot5 (*N_u_*) was calculated by:

*N_u_* = $\frac{c_{u}}{c_{Pdots}}$

where *c_u_* is the molar concentration of luminescent unit in TEA-Pdots5, *c_Pdots_* is the molar concentration of TEA-Pdots5. Based on the synthesis procedure of TEA-Pdots, the mass concentration of TEA-P5 in TEA-Pdots5 dispersion was 100 μg·mL^-1^. With a molar mass of 989.4 g·mol^-1^ for a luminescent unit (C₆₉H₇₆N₆), the molar concentration of the luminescent unit (*cᵤ*) was calculated to be 101.1 μM.

The weight-average molecular weight of TEA-P5 was measured to be 21788 Da. Thus, the degree of polymerization (DP), which represents the number of luminescent units per TEA-P5 chain, was calculated to be 22.02. The molar concentration of TEA-P5 in TEA-Pdots5 (*c_polymer_*) was then derived as *c_polymer_* = $\frac{n_{u}}{DP}$ = 4.591 μM.

Assume that each free TEA-P5 is modeled as a cylinder with a radius of *r* and a height of *h*. Based on the widely reported π-π stacking distance (approx. 0.34-0.45 nm) in the field of conjugated polymers and taking into account the van der Waals radii of the side-chain atoms, the *r* of a single TEA-P5 chain was set to 0.5 nm.^[S5]^ Considering each repeating unit of TEA-P5 backbone comprising fluorene and dimethylacridine units, where the length of a fluorene-fluorene repeat unit is 0.83 nm but the boat conformation of dimethylacridine reduces its effective contribution, the length of a luminescent unit was set to 1.5 nm. With a degree of polymerization of 22.02, the *h* of TEA-P5 chain was calculated to be 33.03 nm, yielding a cylinder volume of 25.94 nm^3^. According to the TEM and DLS characterization of TEA-Pdots5 with diameter of 7 nm (Figure S4 and S5), the volume of individual TEA-Pdots5 was 179.6 nm^3^. Thus, the number of TEA-P5 chain per TEA-Pdots5 (*N_chain_*) is approximately 7.

The molar concentration of TEA-Pdots5 (*c_Pdots_*) could be calculated as:

*c_Pdots_* = $\frac{c_{polymer}}{N_{chain}}$ = 0.6559 μM

Consequently, the number of luminescence units per TEA-Pdots5 (*N_u_*) was about 154. Given that each luminescent unit contains two amine groups, it follows that a single TEA-Pdot5 contains a total of 308 amine groups.

**Supporting Figures**


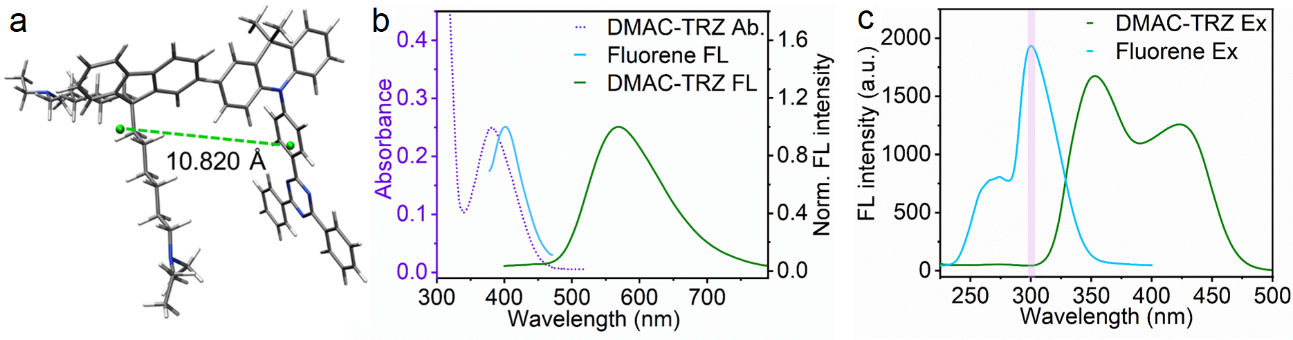


**Figure S1.** (a) Center-to-center distance between donor and acceptor. (b) UV-vis absorption spectrum of DMAC-TRZ, and FL spectra of fluorene and DMAC-TRZ in THF. *λ*_ex_ = 300 nm. (c) FL excitation spectra of fluorene and DMAC-TRZ in THF.


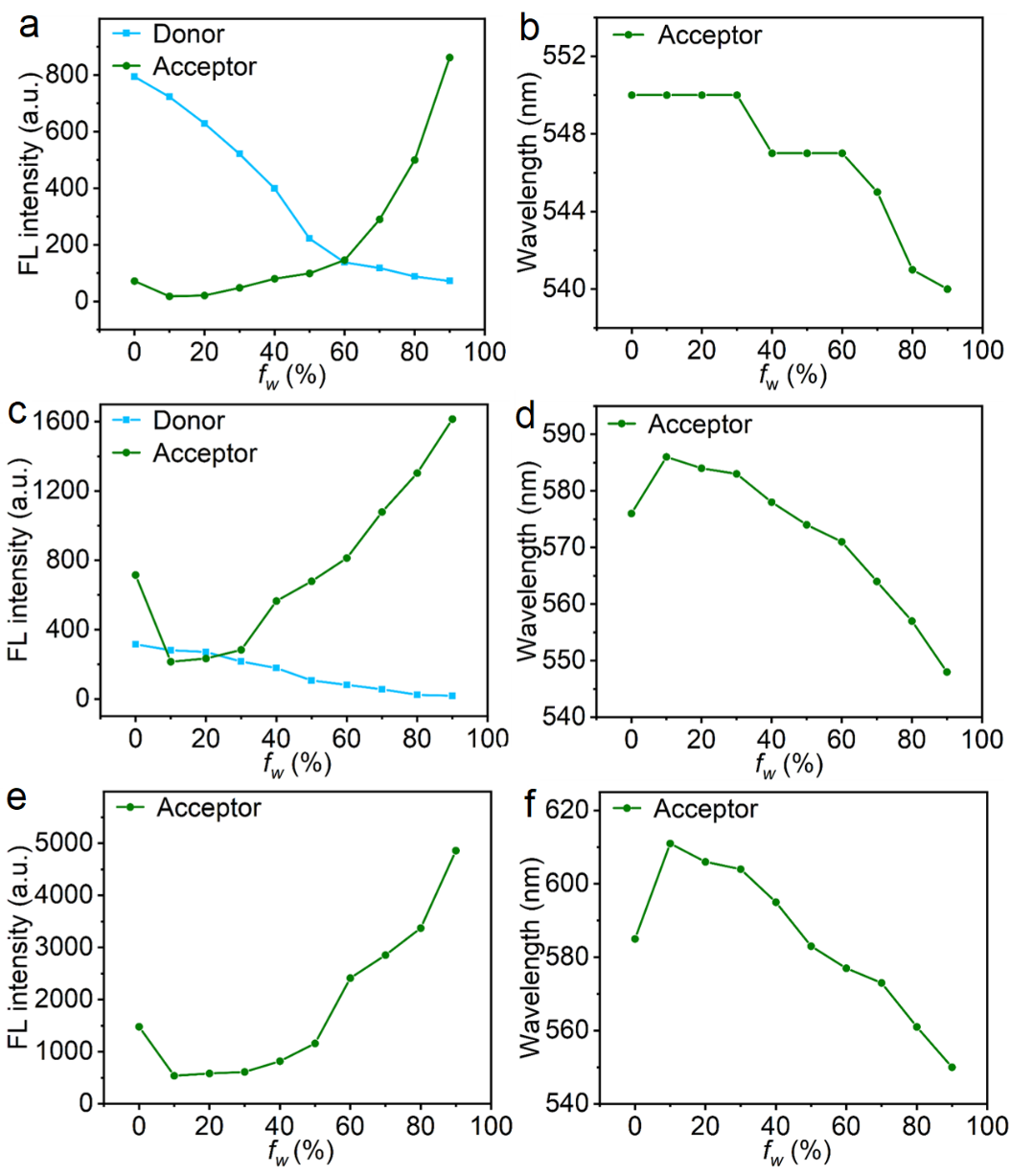


**Figure S2.** Plots of (a, c, e) FL intensity and (b, d, f) wavelength vs *f**_w_* for (a, b) TEA-P1, (c, d) TEA-P3, and (e, f) TEA-P5. *λ*_ex_ = 300 nm.

**
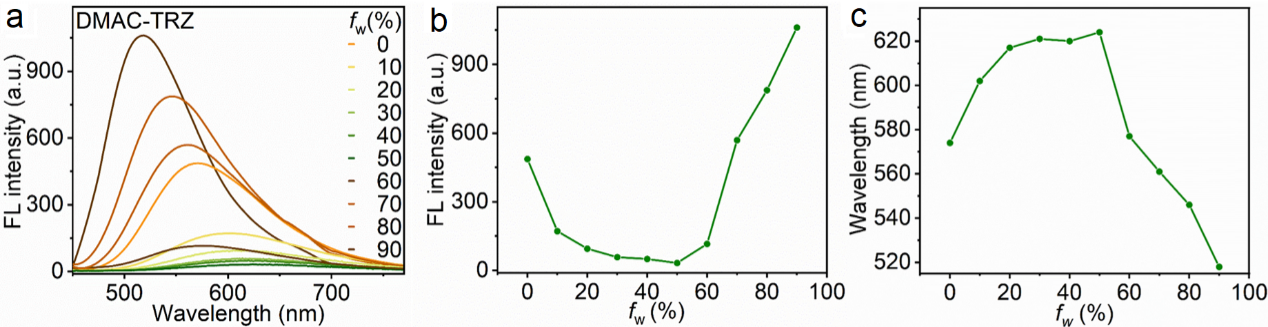
**

**Figure S3**. (a) FL spectra of DMAC-TRZ in THF/water mixture with different water fractions (*f_w_*). (b, c) Plots of FL intensity (b) and wavelength (c) vs *f_w_*. *λ*_ex_ = 365 nm.

**
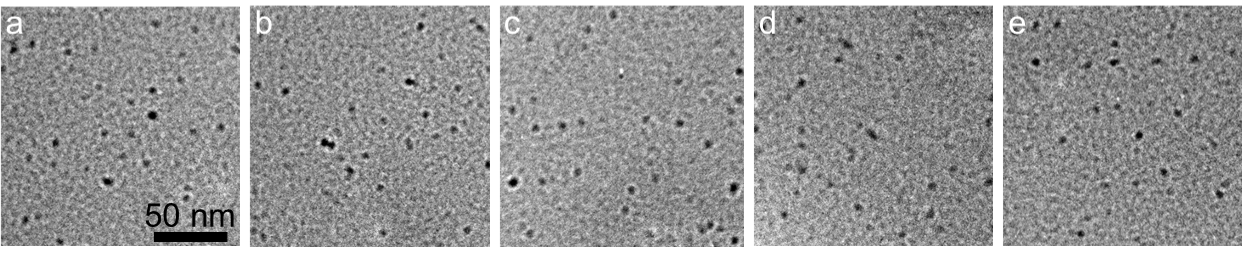
Figure S4**. (a-e) TEM images of TEA-Pdots1~TEA-Pdots5.


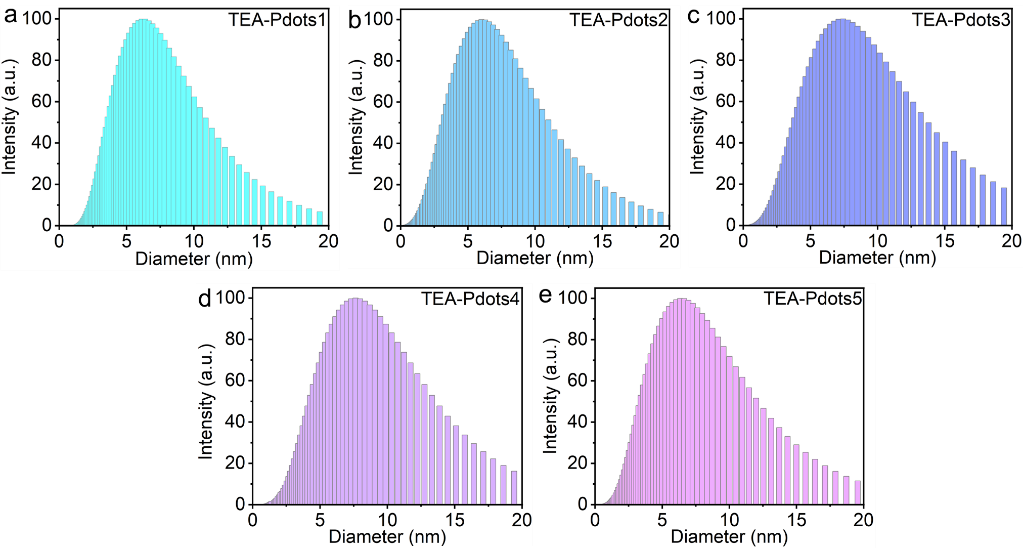


**Figure S5**. (a-e) DLS characterizations of TEA-Pdots1~ TEA-Pdots5.


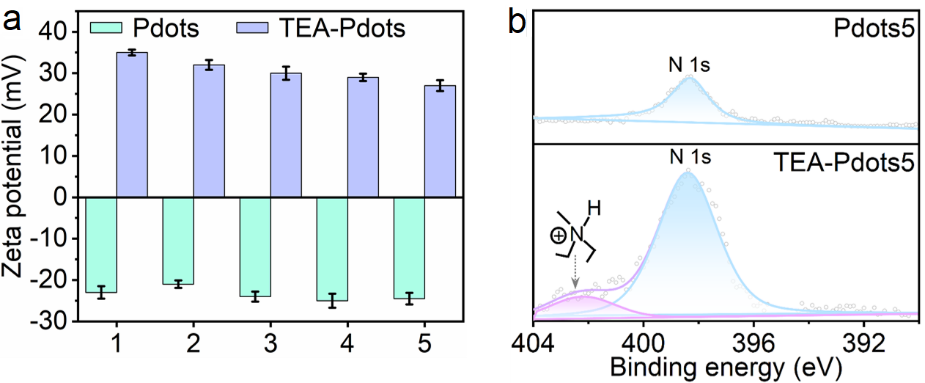


**Figure S****6**. (a) Zeta potentials of Pdots1~Pdots5 and TEA-Pdots1~TEA-Pdots5. (b) N 1s XPS spectra of Pdots5 and TEA-Pdots5. The error bars indicate means ± S.D., *n* = 3.


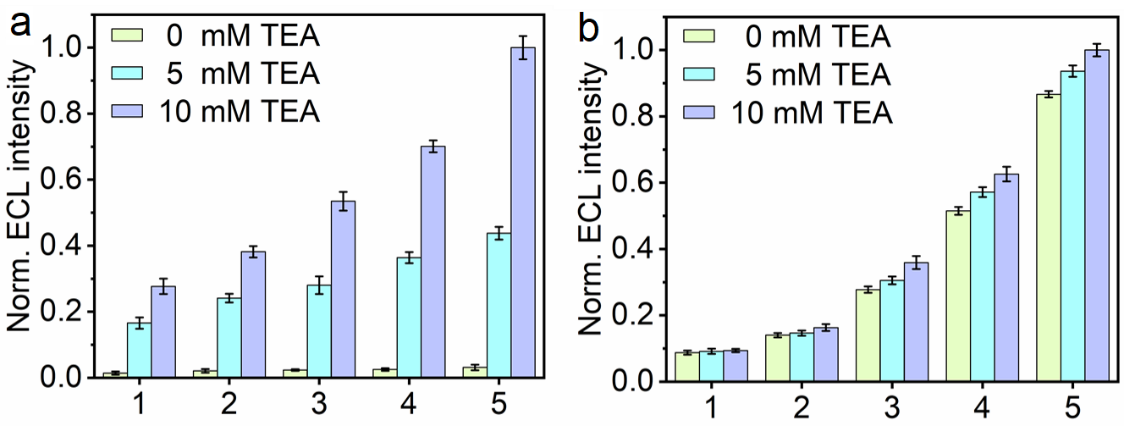


**Figure S7**. Normalized ECL intensity of (a) Pdots1~Pdots5 and (b) TEA-Pdots1~TEA-Pdots5 in 0.1 M PBS containing 0, 5 or 10 mM TEA. The error bars indicate means ± S.D., *n* = 3.


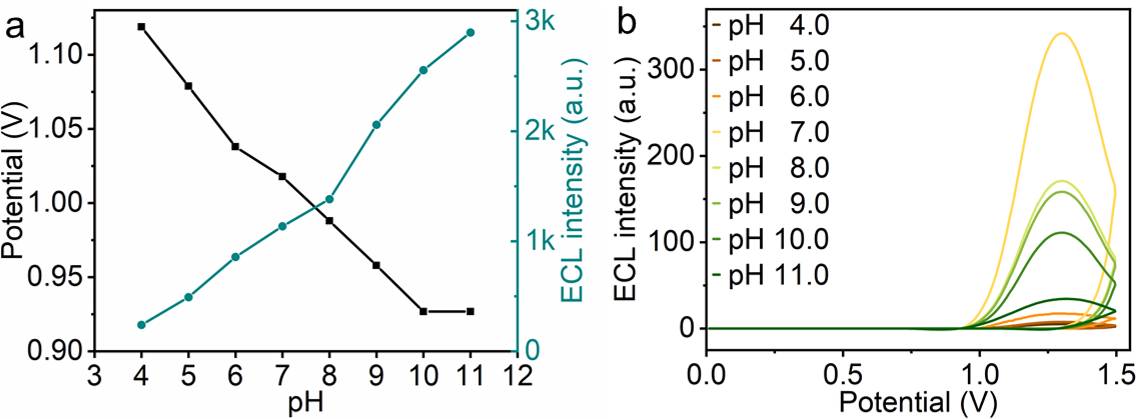


**Figure S8**. (a) ECL peak potentials and ECL intensities of TEA-Pdots5/GCE in 0.1 M PBS with different pHs. (b) ECL curves of Pdots5/GCE in 0.1 M PBS with different pHs containing 10 mM TEA. PMT = 300 V.





**Figure S9.** CV curves of GCE and Pdots5/GCE in 0.1 M PBS.


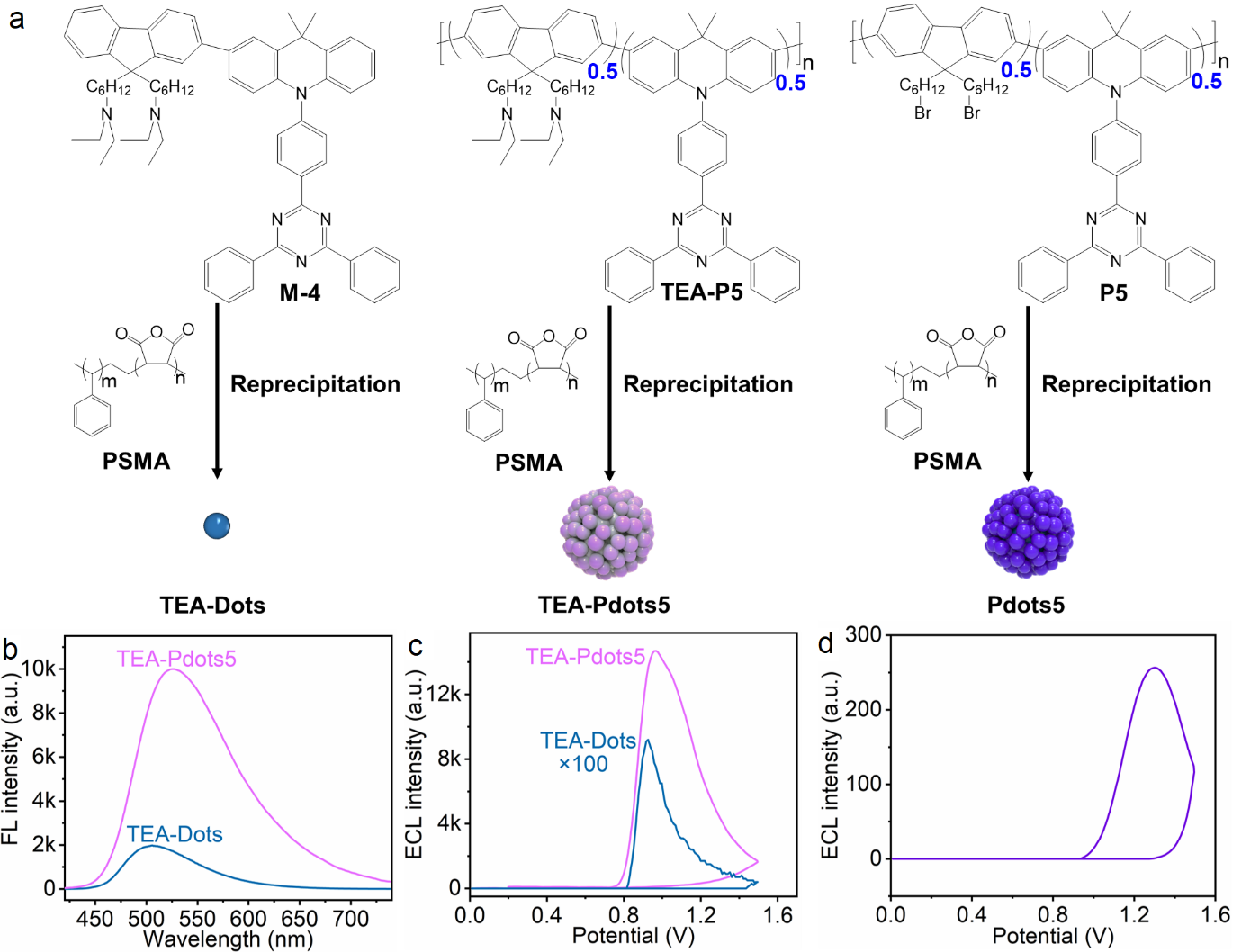


**Figure S10**. (a) Schematic diagrams of TEA-Dots, TEA-Pdots5, and Pdots5 preparation. (b) FL spectra of TEA-Dots and TEA-Pdots5. *λ*_ex_ = 300 nm. (c) ECL curves of TEA-Dots/GCE (after magnified for 100 times) and TEA-Pdots5/GCE in 0.1 M PBS. (d) ECL curve of Pdots5/GCE in 0.1 M PBS containing 10 mM TEA.


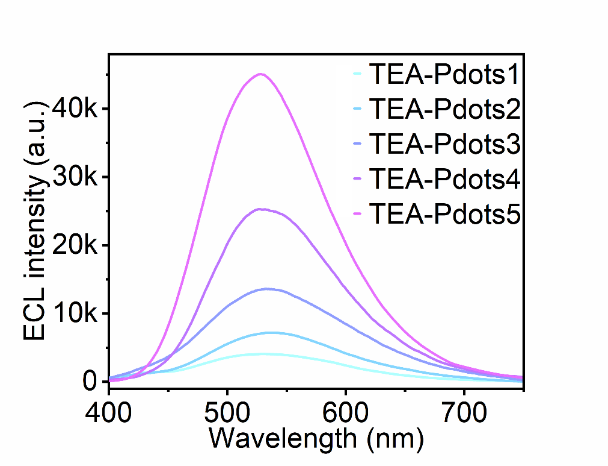


**Figure S11**. ECL spectra of TEA-Pdots1~TEA-Pdots5 in 0.1 M PBS.


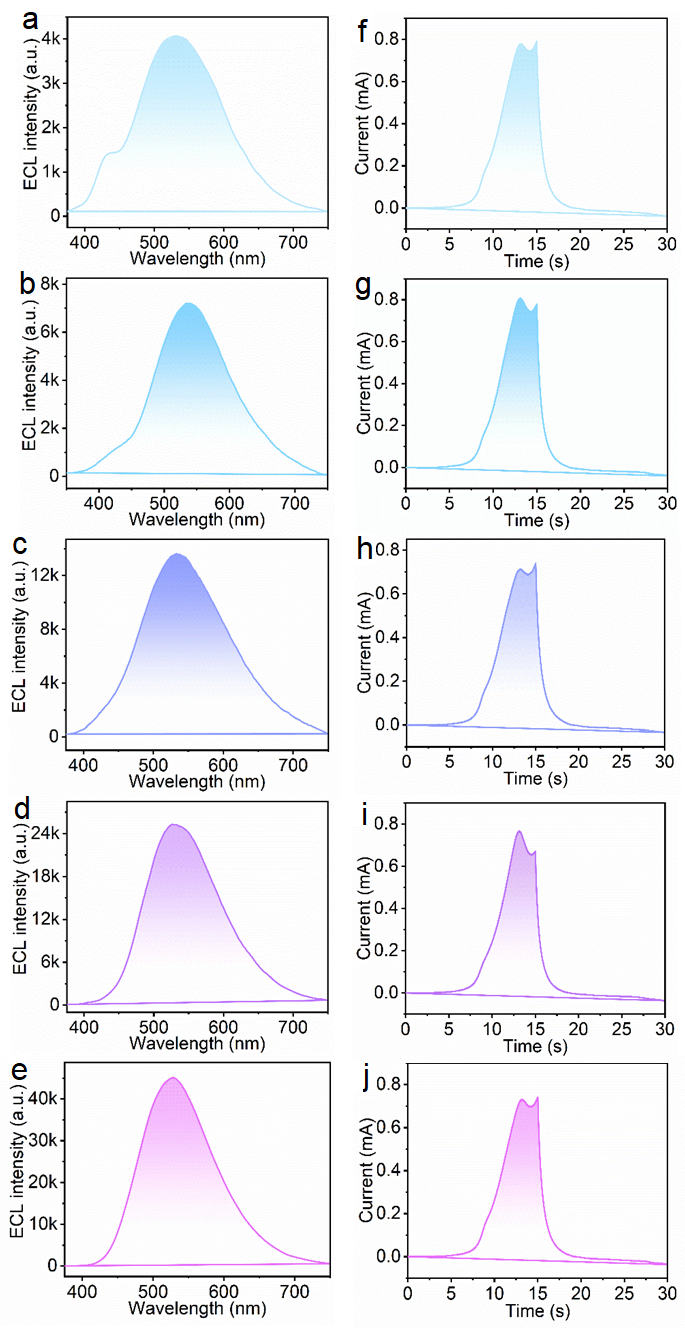


**Figure S12**. (a~e) ECL spectra and (f~j) current-time curves of TEA-Pdots1~TEA-Pdots5 modified GCEs in 0.1 M pH 7.4 PBS.


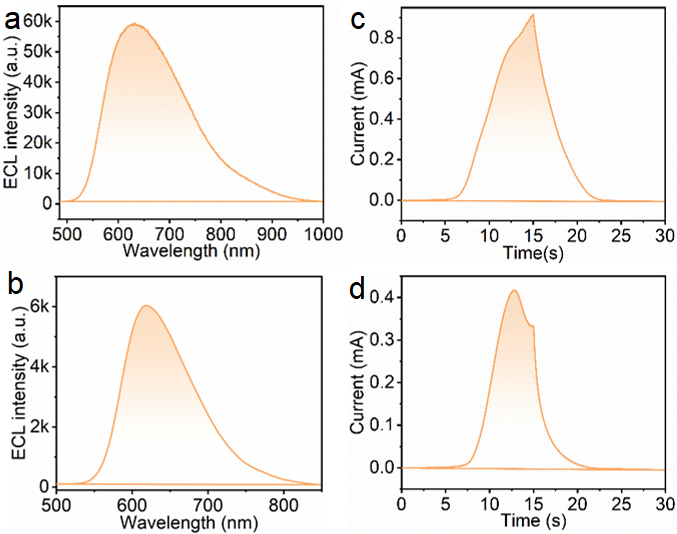


**Figure S13.** (a, b) ECL spectra and (c, d) current-time curves of 10 mM (a, c) and 100 μM (b, d) [Ru(bpy)_3_]^2+^ modified GCEs in 0.1 M pH 7.4 PBS containing 10 mM TPrA (a, c) and 200 μM TEA (b, d).


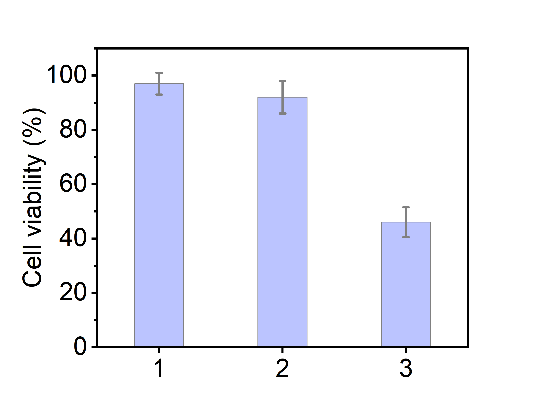


**Figure S14.** MTT analysis of (1) HeLa cells, and (2,3) HeLa cells treated with 50 μg·mL^-1^ TEA-Pdots5 (2), or 10 mM TEA (3). The error bars indicate means ± S.D., *n* = 3.


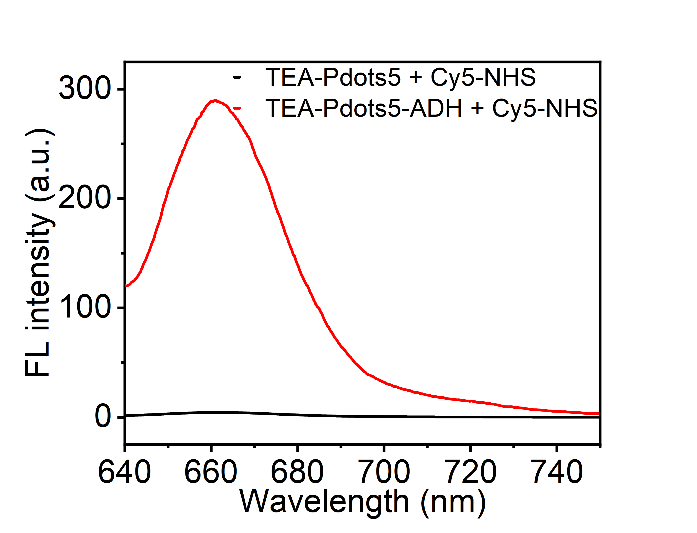


**Figur****e S15.** FL spectra of TEA-Pdots5, and TEA-Pdots5-ADH treated with Cy5-NHS. *λ*_ex_ = 620 nm.


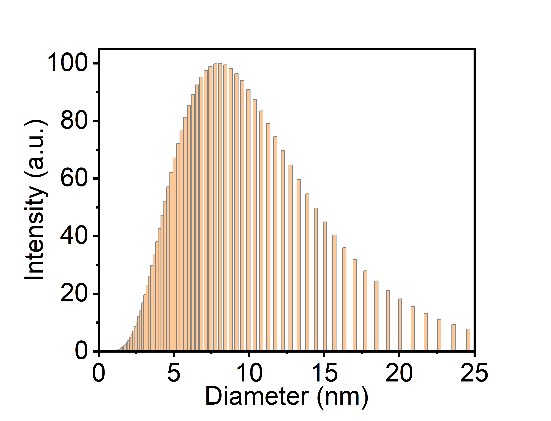


**Figure S16.** DLS characterization of TEA-Pdots5-ADH.

**
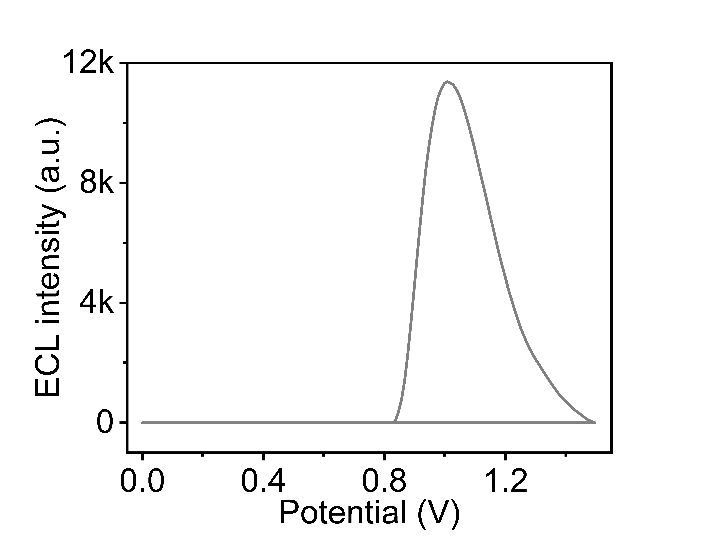
**

**Figure S17.** ECL curve of TEA-Pdots5/ITO in 0.1 M PBS (pH 7.4).


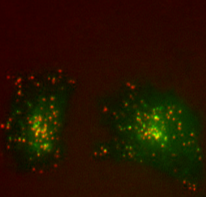


**Figure S18.** Merged image of TIRF (green) and ECL (red) imaging of terminal Gal/GalNAc on HeLa cell.

**Supporting Tables**

**Table S1.** Quantum yields of TEA-P5 in THF/water mixtures with increasing *f_w_*.

| *f_w_* (%) | 0 | 10 | 20 | 30 | 40 | 50 | 60 | 70 | 80 | 90 |
| --- | --- | --- | --- | --- | --- | --- | --- | --- | --- | --- |
| Quantum yield (%) | 5.61 | 1.92 | 1.98 | 2.32 | 3.87 | 8.10 | 26.4 | 41.7 | 55.8 | 78.6 |

**Table S2.** Theoretical vertical excited state energies *ΔE_exc_* (eV) of the first ten singlet and triplet states of TEA-P5 monomer at TD-M062x/6-31G(d, p) level.

| **State** | **S_1_** | **S_2_** | **S_3_** | **S_4_** | **S_5_** | **S_6_** | **S_7_** | **S_8_** | **S_9_** | **S_10_** |
| --- | --- | --- | --- | --- | --- | --- | --- | --- | --- | --- |
| *Δ****E_exc_*** | 2.6030 | 2.9000 | 3.4660 | 3.6030 | 3.6650 | 3.6710 | 3.7220 | 3.7260 | 3.7920 | 3.8320 |
| **State** | **T_1_** | **T_2_** | **T_3_** | **T_4_** | **T_5_** | **T_6_** | **T_7_** | **T_8_** | **T_9_** | **T_10_** |
| *Δ****E_exc_*** | 2.5990 | 2.6160 | 2.8970 | 3.0030 | 3.0990 | 3.1860 | 3.2360 | 3.3590 | 3.5420 | 3.5720 |

**Table S3.** ECL behavior comparison among different Pdots.

| **ECL emitter/coreactant** | **ECL onset potential** | **ECL peak potential** | **Ref.** |
| --- | --- | --- | --- |
| RET+AIE Pdots/TPrA | +0.89 V | +1.12 V | [S6] |
| PFBT-derived TEA-Pdots | +1.00 V | +1.18 V | [S7] |
| Silole Pdots/TPrA | +0.68 V | +1.1 V | [S8] |
| AIE Pdots/TPrA | +0.90 V | +1.16 V | [S9] |
| PFBT NPs+PEDOT/TPrA | +0.78 V | +1.27 V | [S10] |
| TEA-Pdots5 | +0.72 V | +0.96 V | This work |

**Table S4**. Relative ECL efficiency of TEA-Pdots/GCE vs [Ru(bpy)_3_]^2+^/GCE.

| **System** | ***I*/10^5^** | ***Q*/10^-3^** | ***Φ_ECL_*** |
| --- | --- | --- | --- |
| TEA-Pdots1 | 6.61 | 3.87 | 9.79% |
| TEA-Pdots2 | 10.9 | 3.86 | 16.2% |
| TEA-Pdots3 | 21.5 | 3.59 | 34.3% |
| TEA-Pdots4 | 34.9 | 3.43 | 58.2% |
| TEA-Pdots5 | 58.7 | 3.63 | 92.6% |
| 1 mM [Ru(bpy)_3_]^2+^/10 mM TPrA | 115 | 6.59 | 100% |
| 100 μM [Ru(bpy)_3_]^2+^/200 μM TEA | 10.0 | 2.28 | 25.2% |

**Table S5.** ECL efficiency comparison among different ECL nanoemitters.

| **ECL emitter** | **Standard system** | ***Φ_ECL_*** | **Ref.** |
| --- | --- | --- | --- |
| TADF Pdots | 1 mM [Ru(bpy)_3_]/10 mM TPrA | 49.9% | [S11] |
| Hot exciton nanomaterials | 1 mM [Ru(bpy)_3_]/10 mM TPrA | 56.7% | [S12] |
| DMAC-TRZ nanoparticles | 1 mM [Ru(bpy)_3_]/10 mM TPrA | 62.9% | [S13] |
| PFBT-derived TEA-Pdots | 1 mM [Ru(bpy)_3_]/10 mM TEA | 91.0% | [S7] |
| TEA-Pdots5 | 1 mM [Ru(bpy)_3_]/10 mM TPrA | 92.6% | This work |

**Appendix**


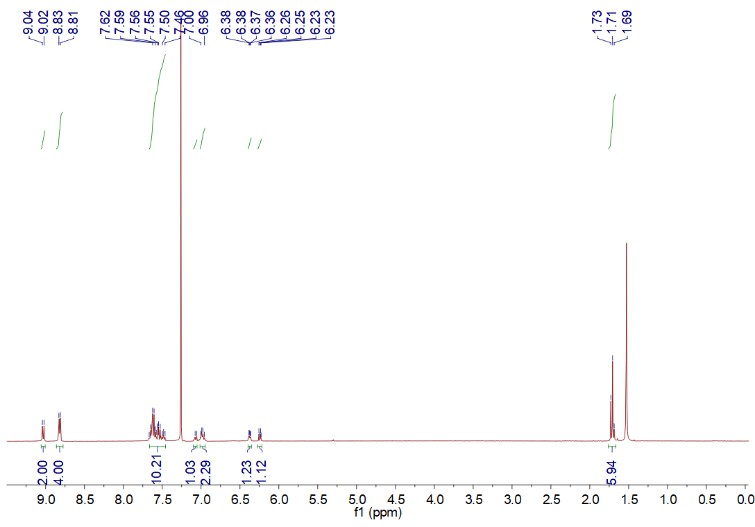


**Appendix S1**. ^1^H NMR spectrum of M-1.


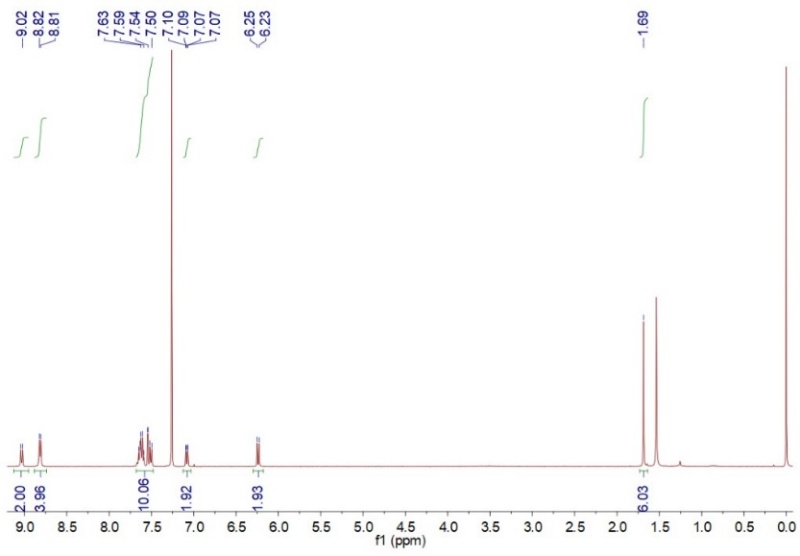


**Appendix S2**. ^1^H NMR spectrum of M-5.


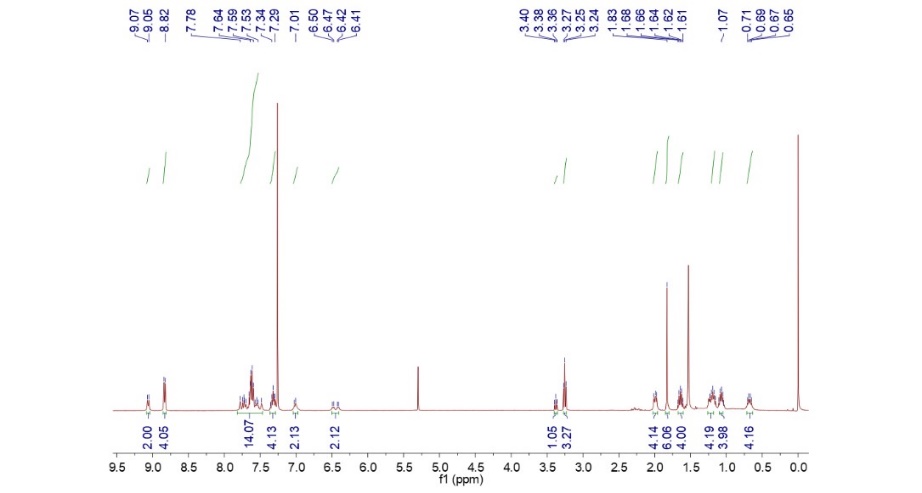


**Appendix S3**. ^1^H NMR spectrum of M-3.


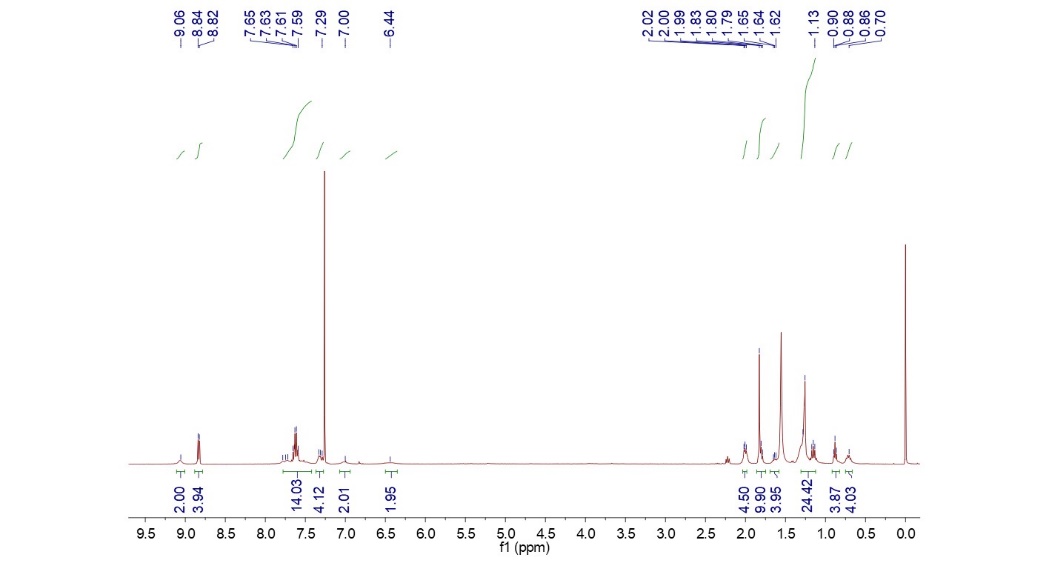


**Appendix S4**. ^1^H NMR spectrum of M-4.


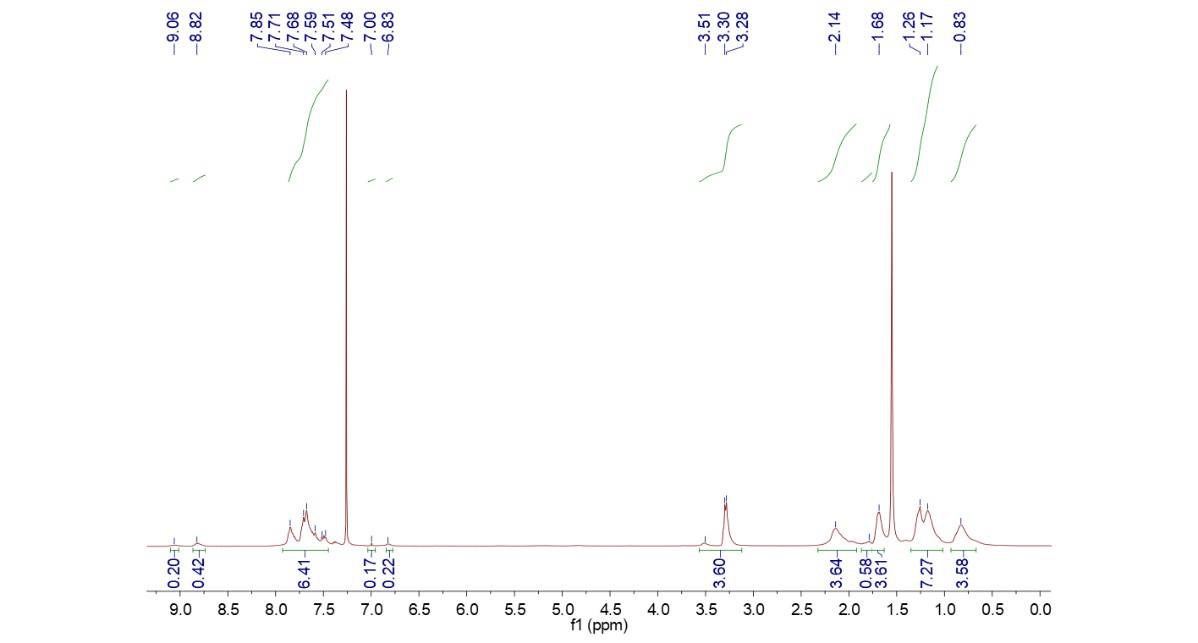


**Appendix S5**. ^1^H NMR spectrum of P1.


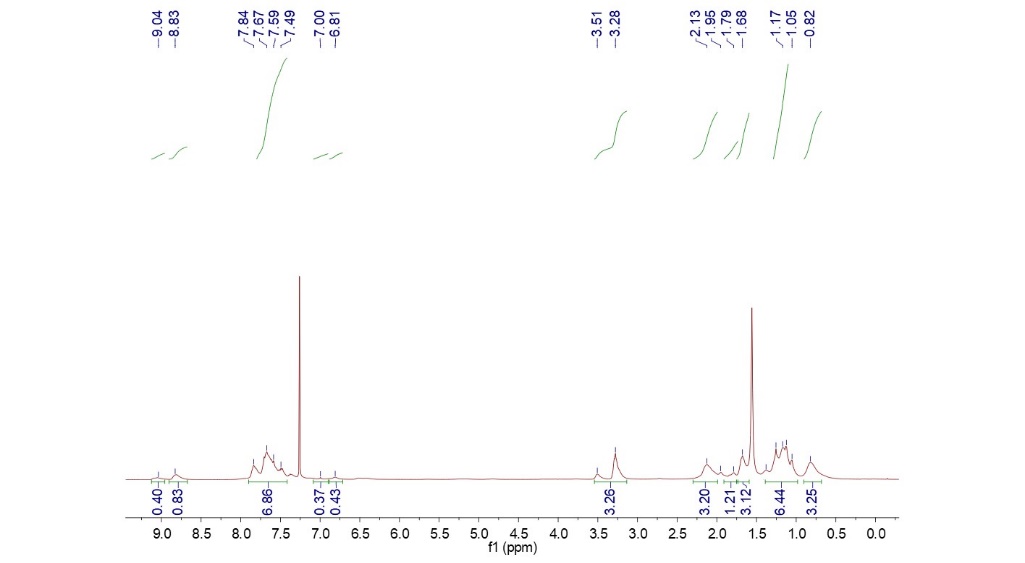


**Appendix S6**. ^1^H NMR spectrum of P2.


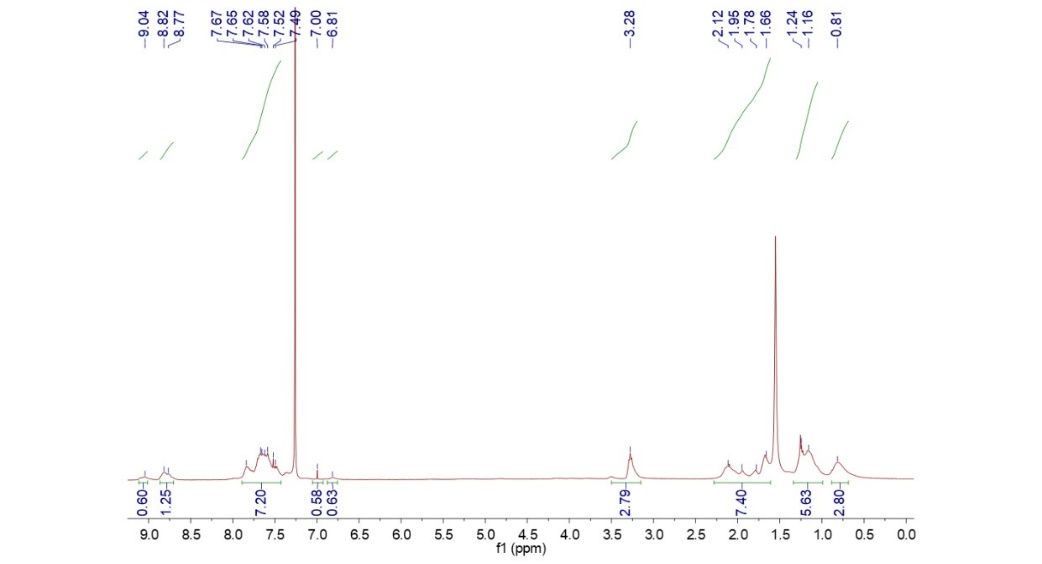


**Appendix S7**. ^1^H NMR spectrum of P3.


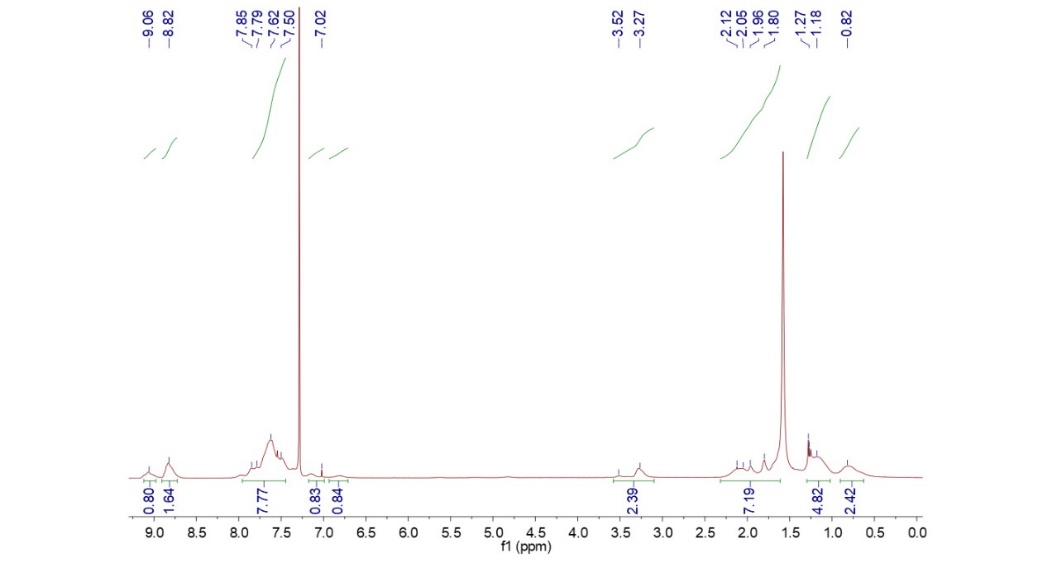


**Appendix S8**. ^1^H NMR spectrum of P4.


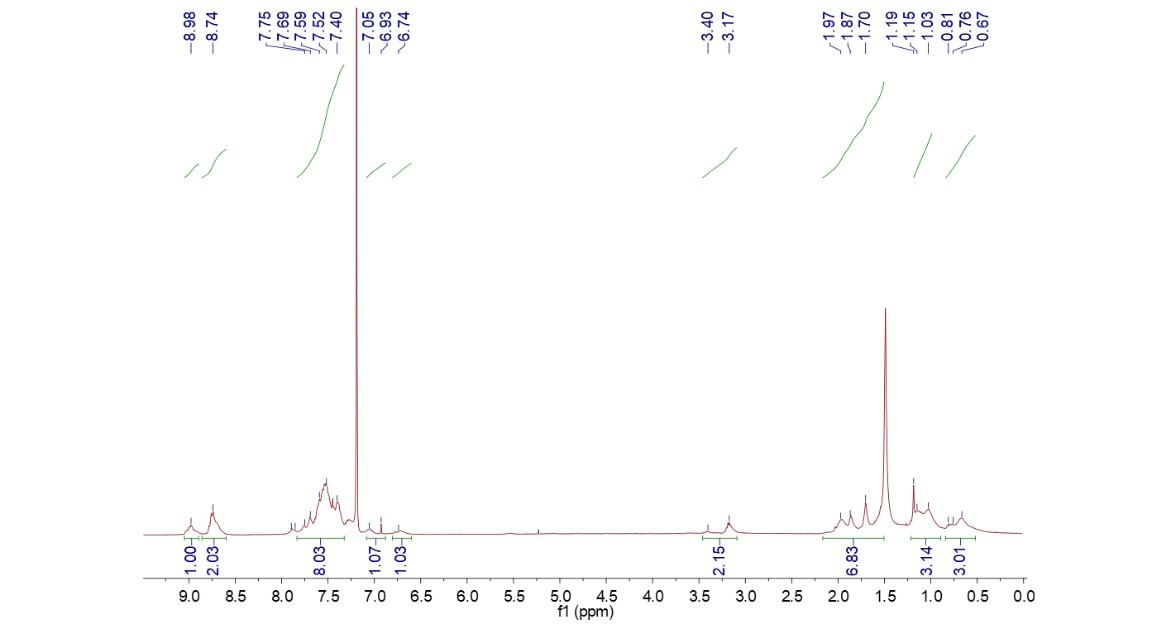


**Appendix S9**. ^1^H NMR spectrum of P5.


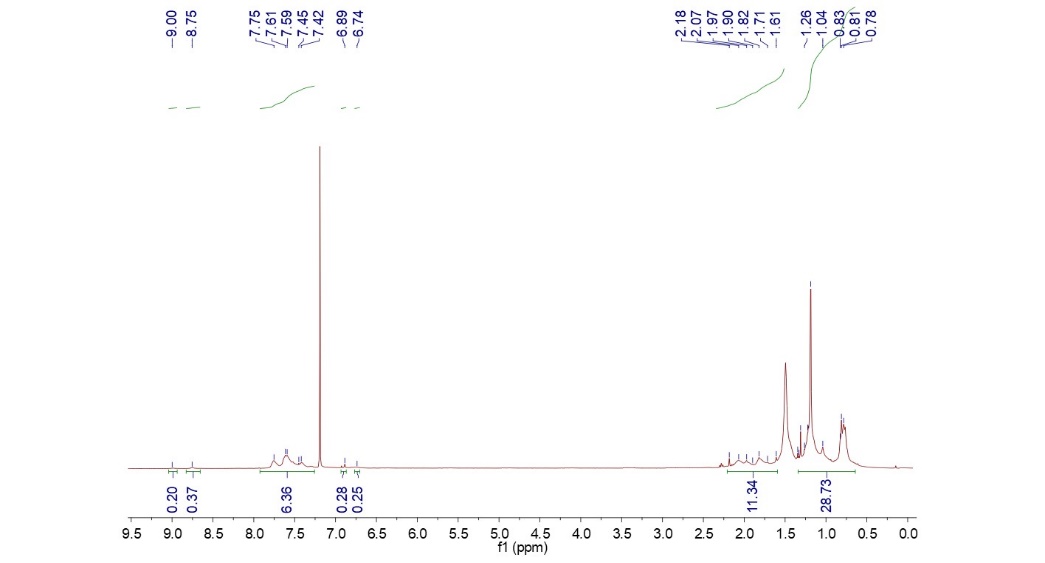


**Appendix S10**. ^1^H NMR spectrum of TEA-P1.


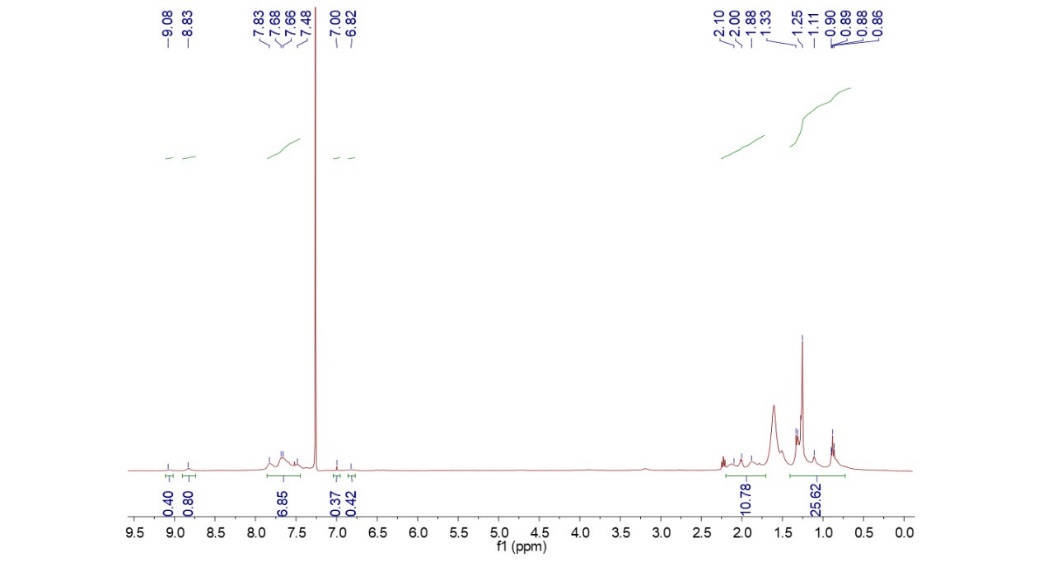


**Appendix S11**. ^1^H NMR spectrum of TEA-P2.


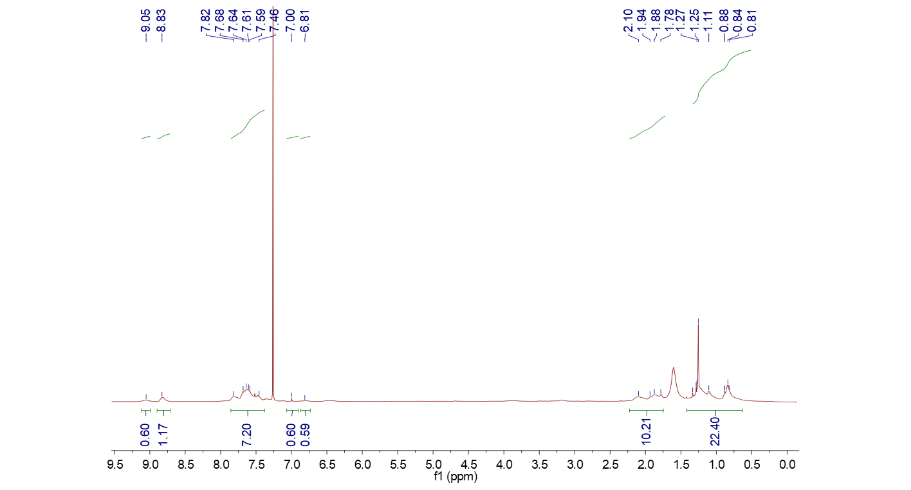


**Appendix S12**. ^1^H NMR spectrum of TEA-P3.


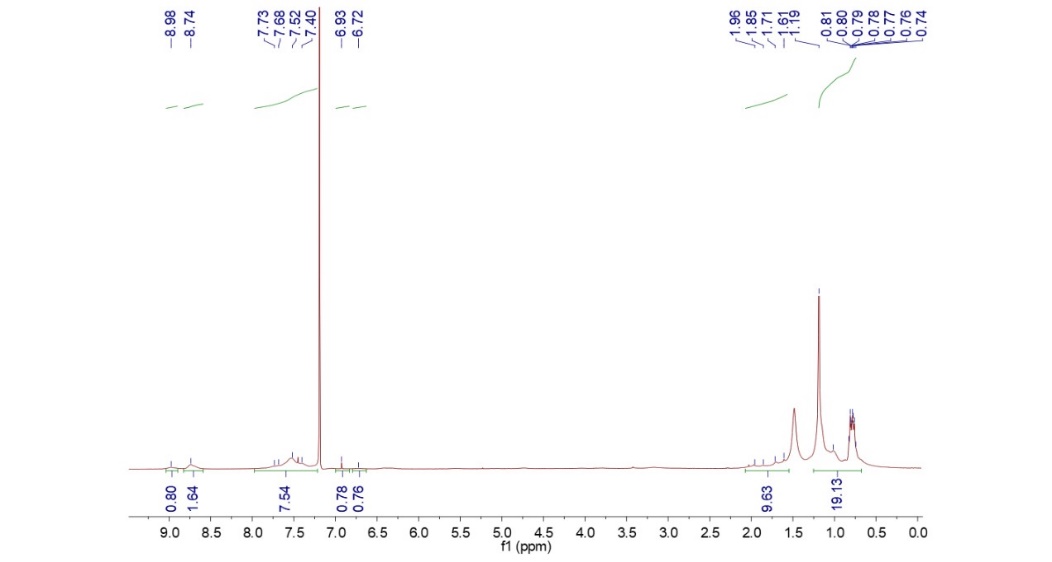


**Appendix S13**. ^1^H NMR spectrum of TEA-P4.


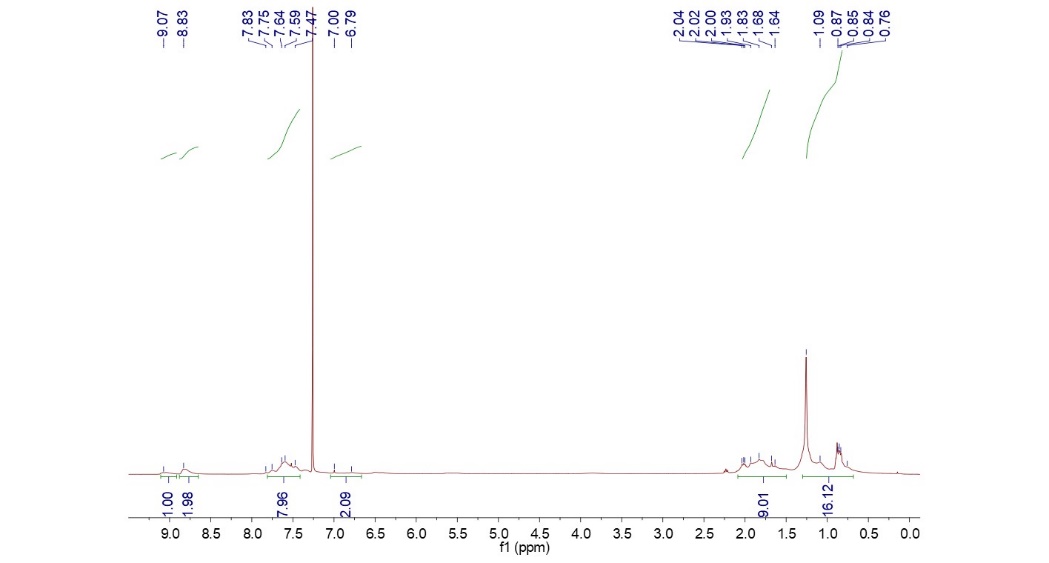


**Appendix S14**. ^1^H NMR spectrum of TEA-P5.

**References**

S1. T. A. Lin, T. Chatterjee, W. L. Tsai, et al., “Sky-Blue Organic Light Emitting Diode with 37% External Quantum Efficiency Using Thermally Activated Delayed Fluorescence from Spiroacridine-Triazine Hybrid,” *Advanced Materials* 28, (2016): 6976.

S2. M. J. Frisch, G. W. Trucks, H. B. Schlegel, et al., Gaussian 16 Revision. A.03, Gaussian Inc., Wallingford, CT, (2016).

S3. T. Lu, and F. Chen, “Multiwfn: A Multifunctional Wavefunction Analyzer,” *Journal of Computational Chemistry* 33, (2012): 580.

S4. M. Godumala, S.Choi, H. J. Kim, et al., “Novel Dendritic Large Molecules as Solution-Processable Thermally Activated Delayed Fluorescent Emitters for Simple Structured Non-doped Organic Light Emitting Diodes,” *Journal of Materials Chemistry C* 6, (2018): 1160.

S5. Y. Q. Feng, F. Sun, N. N. Wang, J. P. Lei, and H. X. Ju, “Ru(bpy)_3_^2+^ Incorporated Luminescent Polymer Dots: Double Enhanced Electrochemiluminescence for Detection of Single Nucleotide Polymorphism,” *Analytical Chemistry* 89, (2017): 7659.

S6. N. N. Wang, Z. Y. Wang, L. Z. Chen, et al., “Dual Resonance Energy Transfer in Triple Component Polymer Dots to Enhance Electrochemiluminescence for Highly Sensitive Bioanalysis,” *Chemical Science* 10, (2019): 6815.

S7. N. N. Wang, H. Gao, Y. Z. Li, et al., “Dual Intramolecular Electron Transfer for In Situ Coreactant-Embedded Electrochemiluminescence Microimaging of Membrane Protein,” *Angewandte Chemie International Edition* 60, (2021):197.

S8. Y. Q. Feng, C. H. Dai, J. P. Lei, H. X. Ju, and Y. X. Cheng, “Silole-Containing Polymer Nanodot: An Aqueous Low-Potential Electrochemiluminescence Emitter for Biosensing,” *Analytical Chemistry* 88, (2016): 845.

S9. M. M. Chen, H. Gao, Z. B. Ge, F. J. Zhao, J. J. Xu, and P. Wang, “Ultrasensitive Electrochemiluminescence Sensor Utilizing Aggregation-Induced Emission Active Probe for Accurate Arsenite Quantification in Rice Grains,” *Journal of Agricultural and Food Chemistry* 72, (2024): 2826.

S10. Y. He, J. W. Zhao, G. M. Yang, R. Yuan, and S. H. Chen, “Interfacial Conductor-Modulated Low-Triggered Potential Electrochemiluminescence from Conjugated Polymers for Bioanalysis,” *Analytical Chemistry* 96, (2024): 17377.

S11. C. Wang, J. Wu, H. Huang, Q. Q. Xu, and H. X. Ju, “Electrochemiluminescence of Polymer Dots Featuring Thermally Activated Delayed Fluorescence for Sensitive DNA Methylation Detection,” *Analytical Chemistry* 94, (2022): 15695.

S12. C. Wang, L. J. Cui, J. Wu, et al., “Electrochemiluminescence of Hot Exciton Nanomaterial with Boosted Efficiency for Visual Bioanalysis,” *Nano Today* 54, (2024): 1021.

S13. C. Wang, Z. W. Tang, Y. R. Li, M. J. Li, H. J. Xie, and H. X. Ju, “Aggregation of Thermally Activated Delayed Fluorescence Molecules to Boost Solid Electrochemiluminescence Efficiency for Biosensing of Protein-Specific Glycoform,” *Science China Chemistry* 68, (2025): 3826.
